# Quantum-enhanced diamond molecular tension microscopy for quantifying cellular forces

**DOI:** 10.1126/sciadv.adi5300

**Published:** 2024-01-24

**Authors:** Feng Xu, Shuxiang Zhang, Linjie Ma, Yong Hou, Jie Li, Andrej Denisenko, Zifu Li, Joachim Spatz, Jörg Wrachtrup, Hai Lei, Yi Cao, Qiang Wei, Zhiqin Chu

**Affiliations:** ^1^ College of Polymer Science and Engineering, State Key Laboratory of Polymer Materials and Engineering, Sichuan University, Chengdu 610065, China.; ^2^ Department of Electrical and Electronic Engineering, The University of Hong Kong, Pok Fu Lam, Hong Kong, China.; ^3^ College of Biomass Science and Engineering, Sichuan University, Chengdu 610065, China.; ^4^ 3rd Institute of Physics, Research Center SCoPE and IQST, University of Stuttgart, 70569 Stuttgart, Germany.; ^5^ National Engineering Research Center for Nanomedicine, College of Life Science and Technology, Huazhong University of Science and Technology, Wuhan 430074, China.; ^6^ Department for Cellular Biophysics, Max Planck Institute for Medical Research, Jahnstraße 29, 69120 Heidelberg, Germany.; ^7^ Institute for Molecular Systems Engineering and Advanced Materials (IMSEAM), University of Heidelberg, Im Neuenheimer Feld 225, 69120 Heidelberg, Germany.; ^8^ Max Planck Institute for Solid State Research, Stuttgart, Germany.; ^9^ National Laboratory of Solid State Microstructures, Department of Physics, Nanjing University, Nanjing 210093, China.; ^10^ School of Biomedical Sciences, The University of Hong Kong, Pok Fu Lam, Hong Kong, China.; ^11^ Advanced Biomedical Instrumentation Centre, Hong Kong Science Park, Shatin, New Territories, Hong Kong, China.

## Abstract

The constant interplay and information exchange between cells and the microenvironment are essential to their survival and ability to execute biological functions. To date, a few leading technologies such as traction force microscopy, optical/magnetic tweezers, and molecular tension–based fluorescence microscopy are broadly used in measuring cellular forces. However, the considerable limitations, regarding the sensitivity and ambiguities in data interpretation, are hindering our thorough understanding of mechanobiology. Here, we propose an innovative approach, namely, quantum-enhanced diamond molecular tension microscopy (QDMTM), to precisely quantify the integrin-based cell adhesive forces. Specifically, we construct a force-sensing platform by conjugating the magnetic nanotags labeled, force-responsive polymer to the surface of a diamond membrane containing nitrogen-vacancy centers. Notably, the cellular forces will be converted into detectable magnetic variations in QDMTM. After careful validation, we achieved the quantitative cellular force mapping by correlating measurement with the established theoretical model. We anticipate our method can be routinely used in studies like cell-cell or cell-material interactions and mechanotransduction.

## Teaser

An innovative quantum-enhanced diamond molecular tension microscopy has been developed to precisely quantify the cellular forces.

## INTRODUCTION

Biochemical factors in the environment are known to affect living organisms and have been investigated for quite a long time (*1*, *2*). Recent evidence has also shown that physical cues such as mechanical forces can constantly be generated inside biological systems and get transmitted to their surroundings (*3*–*5*). The involved mechanical information not only results in deformation and motion but also stimulates physiological functions of lives (*6*–*8*). Normally, the forces associated with a single cell can range from piconewtons to several nanonewtons, corresponding to molecular and cellular levels, respectively (*9*, *10*). In this regard, inventing a reliable tool to quantify the mechanical interactions between the cell and substrate, especially at the single cellular level, is crucial for our basic understanding of many important biological processes such as morphogenesis, tissue repair, and tumor metastasis (*5*, *11*).

Various methods have been successfully developed for measuring cellular adhesive forces in the past few decades. In general, these approaches can be divided into three categories: (i) The first type relies on monitoring the deformation of the substrate to estimate the force, with prime examples being the so-called cellular traction force microscopy (TFM) and micropillar-based force measuring apparatus (*12*); (ii) the second category is the single-cell force spectroscopy by using an instrument like atomic force microscopy (AFM) or magnetic/optical tweezer systems (*13*); and (iii) the third kind is the molecular tension–based fluorescence microscopy (MTFM) or similar tension gauge tether (TGT) systems with the help of force-sensitive fluorophores (*14*, *15*). Although these techniques have been well established as standard tools in mechanobiology study, several issues have also been raised during their implementation in actual cellular measurements. For example, the intrinsic experimental caveats of conventional TFM are known to be computationally intensive and, furthermore, such a method can mainly sense the shear tractions at the nanonewton level (*16*, *17*). In addition, the MTFM and TGT suffer from photobleaching of fluorophores with a stochastic nature (*18*, *19*). Therefore, the development of a technique to accurately measure the cell adhesive forces, preferred in a fluorescent label-free manner, is vital to the development of mechanobiology.

The nitrogen-vacancy (NV) centers, a kind of photoluminescent defect in diamond, display a number of attractive features, including unlimited photostability, unique spin properties with optical readout, chemical inertness, flexible modalities, and excellent biocompatibility (*20*–*22*). Specifically, the electronic spin–dependent photoluminescence associated with a negatively charged NV center (NV^−^) can facilitate optical readout of various physical quantities [e.g., magnetic fields (*23*), electric fields (*24*), and temperature (*25*)] at ambient conditions via conventional fluorescence microscopy, namely, quantum sensing. The NV-based nanoscale quantum sensors, hosted in biocompatible and robust diamond materials, show great promise for applications ranging from fundamental to applied sciences (*26*–*28*). In particular, a set of promising biosensors have been developed in recent years, including intracellular thermometers (*29*–*33*), intracellular orientation tracking agents (*34*, *35*), intracellular free radicals detectors (*36*–*40*), monitoring of physiological species (*41*–*43*), detection of neuronal action potential (*44*, *45*), and magnetic imaging of biomolecules (*46*–*54*). Despite all these exciting progresses, to the best of our knowledge, the direct sensing of weak mechanical signals in living systems has never been achieved yet.

**Fig. 1.**
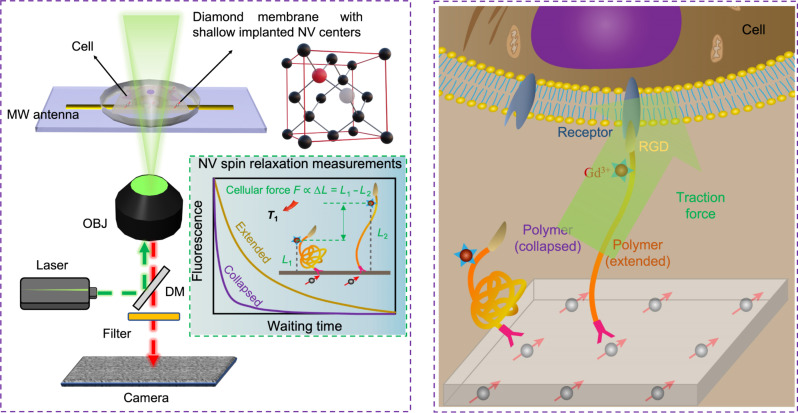
Schematic diagram illustrating the design of QDMTM. **Left:** The working principle of the widefield quantum diamond microscope. The inset shows how the exerted cellular forces can be quantified by measuring NV centers. **Right:** The exact force sensing mechanism. MW antenna, microwave antenna; OBJ, objective; DM, dichroic mirror.

Here, by combining next-generation quantum measurement platforms, with innovative biointerface engineering technologies, we have developed an effective method, termed quantum-enhanced diamond molecular tension microscopy (QDMTM) for the accurate measurement of cellular adhesion forces (Fig. 1). By coupling mechanical signals to the fluorescence of NV centers, the minute cellular forces induced changes of the polymer will be properly quantified through well-established quantum sensing protocols. We experimentally verified the QDMTM in a series of carefully designed control experiments and further showcased the semiquantitative/quantitative mapping of cellular forces at the single cellular level. Our experimental results agreed well with theoretical calculations, suggesting that the proposed platform could be, in principle, upgraded into a standardized toolkit.

## RESULTS

### Construction of a robust quantum diamond biosensing platform

The key of our sensing platform, namely, the QDMTM, was to correlate (as shown in Fig. 1) NV spin relaxometry with force-induced polymer stretching, which was documented using the well-known worm-like chain (WLC) model (*55*). In the case where the magnetic labels (Gd^3+^ ions) were attached to the diamond surface through a spring-like polymer, the relationship between the relaxation rate Γ_1_ and NV-Gd^3+^ distance *h* has been known to obey the following relationship (*56*)

$$\Gamma_{1}\propto h^{-3}$$ (1)

**Fig. 2.**
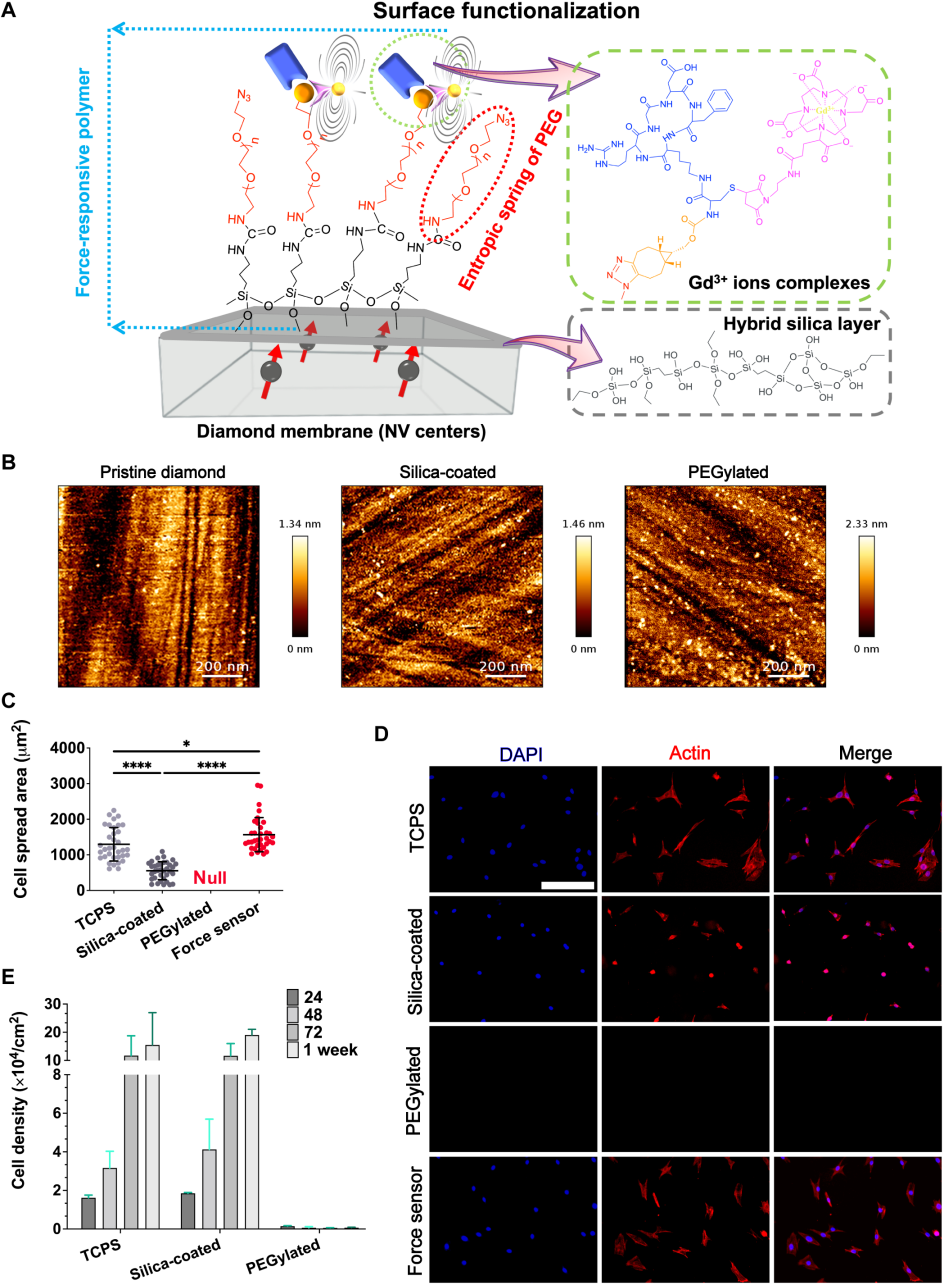
Diamond-based quantum sensing platform. (**A**) Schematic illustration of overall chemical functionalization architecture. (**B**) AFM characterization of the pristine diamond, silica-coated, and PEGylated surfaces of the diamond sensor. (**C**) Cell spreading area [*n* = 40, three technical replicates, *P* values were obtained by one-way analysis of variance (ANOVA) followed by Tukey’s post hoc test, mean with SD] and (**D**) representative enlarged images of NIH 3T3 stained with the cytoskeleton (phalloidin, red) and nuclei [4′,6-diamidino-2-phenylindole (DAPI), blue] after culturing for 16 hours. Scale bar, 100 μm. (**E**) Attachment density of cells on diamond surfaces with different functionalization architectures after incubation for 1, 2, 3, and 7 days (means with SD). TCPS, tissue culture polystyrene.

We adopted our previously developed single-crystalline ultrathin diamond membrane (~30 μm) with shallow implanted NV centers to work as a widefield quantum sensing substrate. To enable the NV-based measurement of the integrin-based cell adhesive force, such mechanical signals can be converted to magnetic ones using a transducer, i.e., the tailor-made force-responsive polymer (Fig. 2A and figs. S1 and S2). Specifically, it contained an integrin ligand Cyclo(RGDfK) (RGD, arginine-glycine-aspartic acid) and a paramagnetic molecule Gd^3+^ on one side, the main molecular chain polyethylene glycol (PEG) serving as the spring element in the middle, and a silane anchor on the other end for immobilization (fig. S3). The PEG chain serving an entropic spring was capable of sensing forces on the order of a few tens of piconewtons (*19*) and provided a bioinert background to avoid nonspecific interactions with cells (*57*). The deformation of the PEG chain altered the distance between the Gd^3+^ ions and the NV centers, leading to the change of NV spin relaxation time *T*_1_ (1/Γ*_1_*) which can be quantitatively measured (*43*).

Anchoring these force-responsive polymers within the effective sensing range of NV centers [~25 nm above the diamond surface (*58*)], meanwhile, minimizing the thickness of any functionalization layer while retaining excellent surface morphology and coverage is crucial for the *T*_1_ test. To enable the conjugation of the designed polymers to the chemically inert surface of the diamond, we first managed to deposit a stable layer of hybrid silica, and such an interface with high reactivity without further activation was sufficient to enhance intra-layer interactions (*59*), and stabilize the PEG coating. Benefitting from this silica layer, the polymer could be easily immobilized onto the diamond surface to construct the desired force-sensing platform in mild conditions to avoid detrimental influence on the spin property of NV centers (fig. S4).

The x-ray photoelectron spectroscopy (XPS) results confirmed the successful conjugation of the force-responsive polymers onto the diamond surface (fig. S5 and table S1). The length of the immobilized PEG polymer was ca. 6 nm in ambient conditions as detected by ellipsometry on the silica surface, while the thickness of a hybrid silica layer was about 2.9 nm (fig. S6). The functional diamond surfaces were analyzed by AFM to confirm the homogeneity of the deposited hybrid silica layer (Rq = 332.1 p.m.) and the polymer coating (Rq = 739.6 p.m.), and the typical aggregates of polymer brushes (*60*) could also be observed (Fig. 2B and fig. S7). These results further confirmed the successful conjugation of customized force-responsive polymers on diamond surfaces. In addition, the immobilized amount of the 2,2′,2″-(10-(4-((2-(3-((3-((4-(14-benzyl-11-(carboxymethyl)-5-(3-guanidinopropyl)-3,6,9,12,15-pentaoxo-1,4,7,10,13-pentaazacyclopentadecan-2-yl)butyl)amino)-2-(((((1R,8S,9s)-bicyclo[6.1.0]non-4-yn-9-yl)methoxy)carbonyl)amino)-3-oxopropyl)thio)-2,5-dioxopyrrolidin-1-yl)ethyl)amino)-1-carboxy-4-oxobutyl)-1,4,7,10-tetraazacyclododecane-1,4,7-triyl)triacetic acid (BCN-RGD-DOTA (BRD)) reached 236 ng/cm^2^ as detected by quartz crystal microbalance (QCM) with dissipation (fig. S8A). This density was sufficient to support cell adhesion and focal adhesion formation (*61*).

The stability of the immobilized polymer on the diamond surface was directly examined in cellular environments. The introduced polymers well supported the adhesion of NIH 3T3 fibroblasts during the whole cell culture period (Fig. 2, C and D, and fig. S9) while preventing the nonspecific adsorption of bovine serum albumin (fig. S8B). Meanwhile, no cells could adhere to the polymer coatings without RGD ligands for at least 7 days (Fig. 2E and fig. S10). These results demonstrated that the polymer coating only interacted with cells through integrin-RGD adhesion and could keep stable for at least several days in cell incubation conditions. This was actually consistent with our reference test with the same polymer brushes on a silicon wafer, i.e., the thickness of the PEG-coated layers (containing hybrid silica layer) kept constant after 5 days of immersing in phosphate-buffered saline (PBS) buffer (fig. S6).

### Validation of quantum-enhanced force sensing

**Fig. 3.**
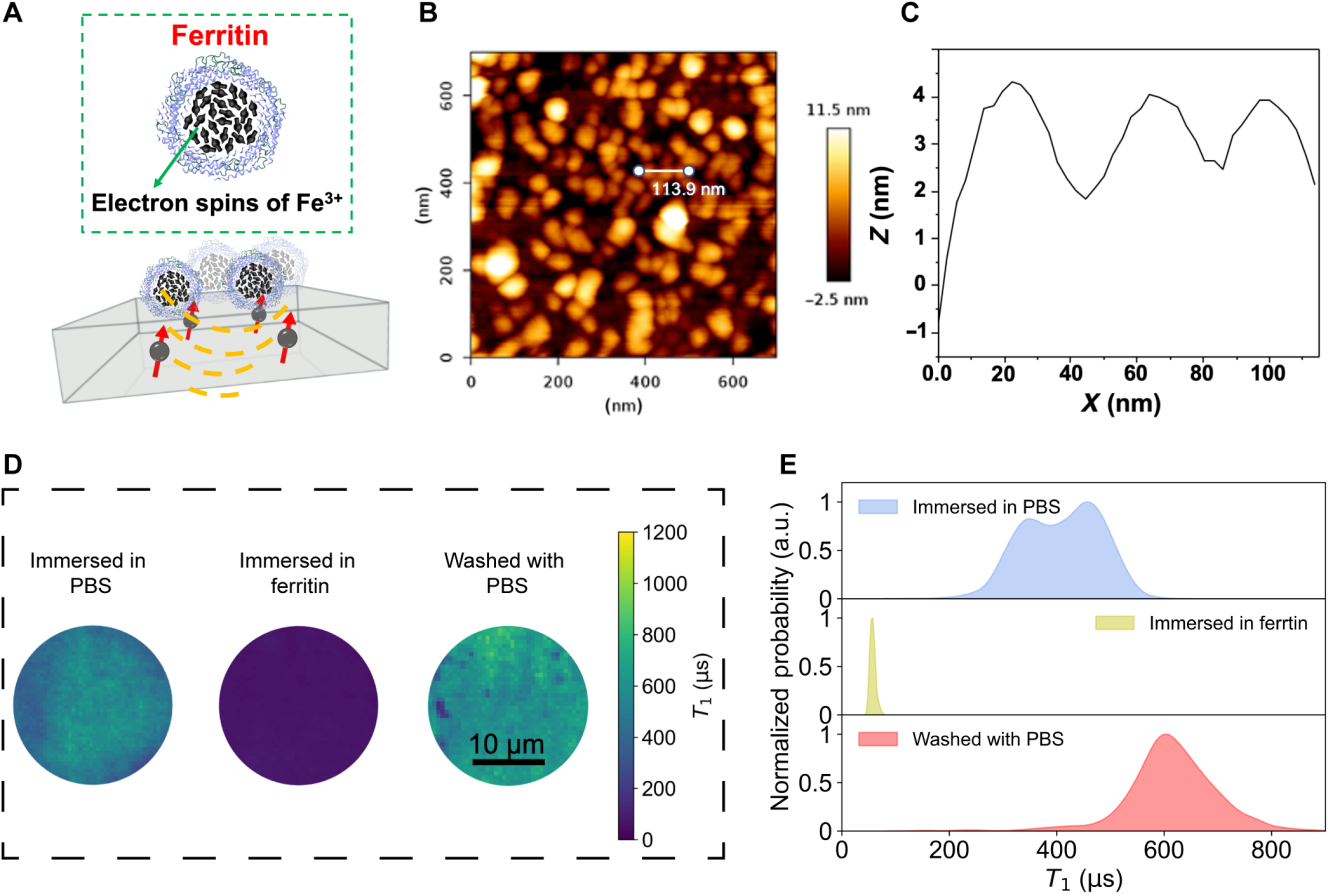
Sensing of magnetically labeled proteins using QDMTM. (**A**) Top: Schematic illustration of ferritin, the black arrows depict the paramagnetic Fe^3+^ ions. Bottom: Schematic diagram of ferritin adsorption on PEGylated surface of diamond membrane. (**B**) AFM image of ferritin adsorbed on PEGylated surface and (**C**) the corresponding height profiles of ferritin adsorbed on PEGylated surface [positions marked in (B)]. (**D**) *T*_1_ mapping image of PEGylated diamond membrane immersed in PBS (left), ferritin (1.0 mg/ml; middle), and washed with PBS afterward (right). (**E**) Corresponding histogram of *T*_1_ mapping in (D). a.u., arbitrary units.

To demonstrate the sensing capability of our customized widefield quantum diamond microscope (detailed in Materials and Methods), we first investigated the influence of ferritin [a kind of paramagnetic species (*62*) as shown in Fig. 3A] solution on the NV spin relaxation in constructed PEGylated sample (without RGD ligands and magnetic labels). As shown in Fig. 3 (B and C), the AFM image clearly indicated that there has been a dense packaged layer of ferritin formed on top of the diamond surface [after immersing in aqueous ferritin solution (1 mg/ml) for 2 hours without rinsing]. In the corresponding *T*_1_ mapping images and histogram (Fig. 3, D, left and middle, and E), we found that the ferritin-deposited diamond surface showed a notably shorter *T*_1_ value (approximately tens of microseconds), i.e., almost one order of magnitude lower than that in PBS cases (approximately hundreds of microseconds). These findings indicated that the presence of ferritin molecules (or gadolinium ions as shown in fig. S11) affects the NV spin relaxation as revealed by *T*_1_ values (*62*). We have actually found that the *T*_1_ value could be recovered after gently washing with PBS (Fig. 3, D, right, and E), indicating that the coated PEG chains successfully prevented the nonspecific adsorption of proteins.

**Fig. 4.**
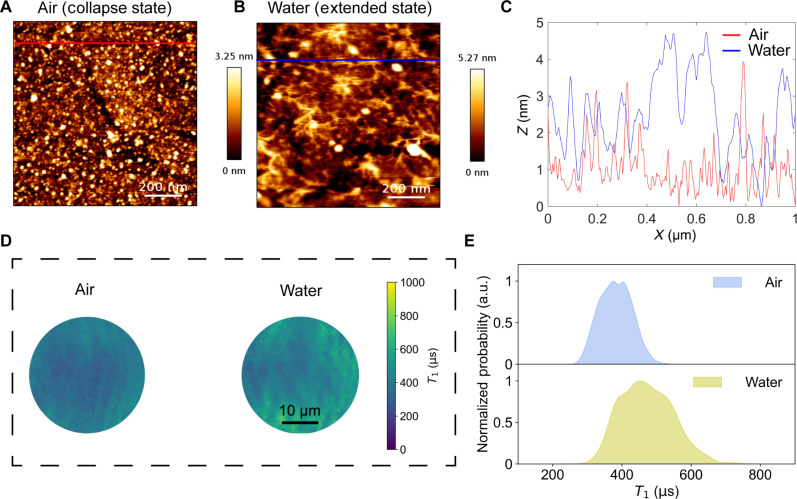
Validation of the designed QDMTM using the polymer conformational changes caused by solvent effects. AFM images of the constructed diamond force sensor in (**A**) air and (**B**) water, respectively. (**C**) Line profile of images shown in (A) and (B). (**D**) *T*_1_ mapping of the constructed diamond sensor in different states. (**E**) Corresponding histogram of *T*_1_ mapping in (D).

To validate the designed QDMTM, we first managed to alter the conformation (collapse-extended) of force-responsive polymers in model conditions, which mimics the polymer chain stretched by cellular forces. Specifically, it is well known that the hydration (e.g., sample immersed in water) could extend the PEG chains, while dehydration (e.g., sample exposed to air) would collapse the PEG chains (*63*). Therefore, the distance between the Gd^3+^ magnetic labels and the NV centers can be modulated by placing the constructed diamond force sensor sample in different environments like water and air. The influence of solvents on the force sensor was confirmed by AFM, which exhibited a relatively homogenous microstructure with a surface roughness of 1198.0 pm for the decorated surfaces (water) (fig. S7). Figure 4 (A and B) shows typical topographies of the polymer-modified diamond surface in air and water, disclosing the morphologies at the collapsed and extended states, respectively (the relative height increases by ~5.27 nm). The corresponding line profiles (positions marked in Fig. 4, A and B) were plotted in Fig. 4C, demonstrating a general tendency for molecular chain stretching caused by solvent effects. In addition, one end of PEG molecules is linked with Gd^3+^ ion complexes and another end is attached to the diamond surface through a very thin “active” layer of hybrid silica. Therefore, extending or collapsing the polymer shell layer changes the NV-Gd^3+^ distance and thus affects the measurement of NV spin relaxation time (*T*_1_) (*43*). From the measured *T*_1_ mapping of the force sensor diamond samples (Fig. 4, D and E), there have been much smaller *T*_1_ values in air (collapsed status: shortened NV-Gd^3+^ distance), compared with that measured in ultrapure water (extended status: prolonged NV-Gd^3+^ distance). The same trend was found to maintain the same even after another cycle (fig. S12, A and B). Thus, the measured *T*_1_ values were highly correlated with NV-Gd^3+^ distance changes modulated by the PEG entropic spring, demonstrating the feasibility of our proposed QDMTM.

### Semiquantitative mapping of cell adhesive forces Γ_1_∝⟨*B*^2^⟩∝*h*^−3^

By seeding the maturely adhered NIH 3T3 cells on a diamond surface modified with force-responsive polymers, we started to demonstrate the detection of cellular adhesion forces using QDMTM. Cell adhesive force is transmitted to the environment through integrin-ligand interactions (*64*), and the force-responsive polymers were used to convert the mechanical input to the magnetic output. As illustrated in Fig. 1, the RGD end of the polymer was assumed to be recognized by integrins and dragged by the cellular traction force. The PEG entropic spring was stretched, and the Gd^3+^ magnetic labels, located just next to the RGD ligands, were moved away from the diamond surface. This distance change could be quantitatively detected via the NV spin relaxometry, i.e., the larger force-induced conformation changes (of polymer) the longer the *T*_1_ value is (of NV centers).

**Fig. 5.**
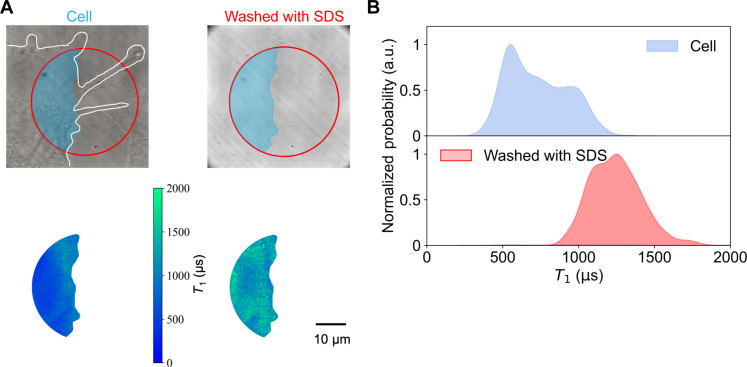
Validation of the designed QDMTM by living cells. (**A**) Top: Transmitted light images of the selected regions in a typical NIH 3T3 cell grown on a diamond force sensor before (left) and after (right) 1% SDS treatment. Bottom: *T*_1_ mapping of the corresponding regions. (**B**) Corresponding histogram of *T*_1_ mapping in the lower panel of (A). White dashed lines indicate the edge of the selected cell.

On the basis of the experimental results above and the theoretical analysis, we verified whether the variation in the distribution of *T*_1_ was correlated with cellular regions. The adhered cells were detached by the treatment with 1% SDS, and the *T*_1_ value of the cell adhered region was recovered to the original level after cell removal (Fig. 5, A and B). This result confirmed that *T*_1_ distribution was influenced by the adherent cells.

**Fig. 6.**
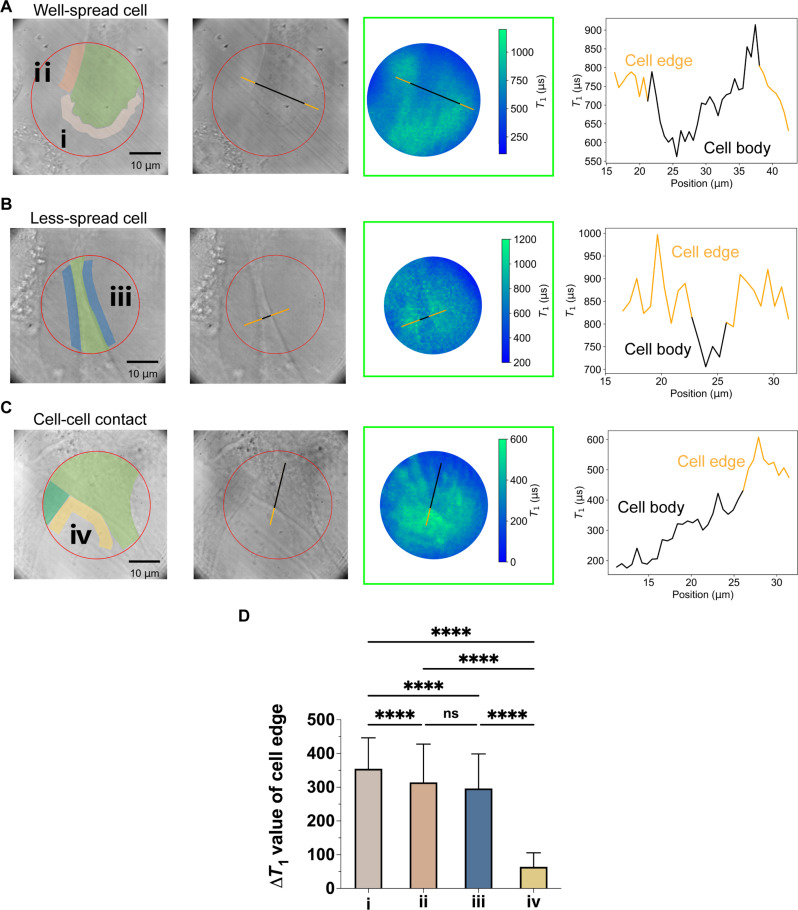
Demonstrated detection of the traction forces of the adhered cells. Three typical cells, namely, the (**A**) well-spread cell, (**B**) less-spread cell, and (**C**) cell-cell contacted cells were chosen to demonstrate the measurements: The first two columns show the transmitted light images and corresponding marked images (left, green represents the cell body, while other colors represent the selected edges of cells); the third column shows the *T*_1_ mapping and the last column shows the line profile, as-marked in the second and third column, of *T*_1_ value across the cell body. (**D**) *T*_1_ values change in selected areas of cells [denoted as i, ii, iii, and iv as shown in (A) to (C), *P* values were obtained by one-way ANOVA followed by Tukey’s post hoc test, means with SD. The detailed calculation of Δ*T*_1_ was described in the experimental section].

Meanwhile, as shown in Fig. 6, A to C (i.e., a few typical examples), the relative *T*_1_ changes (with respect to that measured in water as shown in Fig. 4) were larger in the peripheral area of the cells and sharped near the cell edges, where the pseudopodia and focal adhesions were enriched (fig. S13). This was consistent with the fact that the actomyosin stress fiber, the generator of cell traction force (*65*), mainly connected the two sides of the spread cells, thus the traction force concentrated at the cell edges, especially the edges of the polarized sides (*66*). For well-spread cells, the *T*_1_ values in the polarized region of the cell (Fig. 6A, marked i) are greater than the region of the bridged edges of the cell (Fig. 6A, marked ii) and *T*_1_ values in region ii are similar to those in region iii (Fig. 6D). Meanwhile, the relative *T*_1_ changes in the region of the well-spread cells (Fig. 6, A and D) were larger than that of the less-spread cells (Fig. 6, B and D), indicating the larger adhesive force generated in the well-spread cells. This result matched the previous conclusion that the cell adhesive force was positively related to the cell spread area on the rigid or elastic substrates (*67*). Compared with single-cell measurements (Fig. 6, A and B), fewer *T*_1_ changes were found in measurements with multiple cells (Fig. 6C, marked iv). This was probably due to the cell-cell interaction decreasing the integrin-based adhesive forces, as part of the actomyosin-integrin linkage was disassembled to build the cell-cell adhesion (*68*, *69*). The adhesive region of the less spread cells, which were contacted with neighbor cells (cell-cell contact), showed the lowest *T*_1_ value (Fig. 6D), suggesting the smallest force of integrin adhesion. These experiments further validated the capability of QDMTM in measuring cell adhesive forces.

### Quantitative mapping of cell adhesive forces

**Fig. 7.**
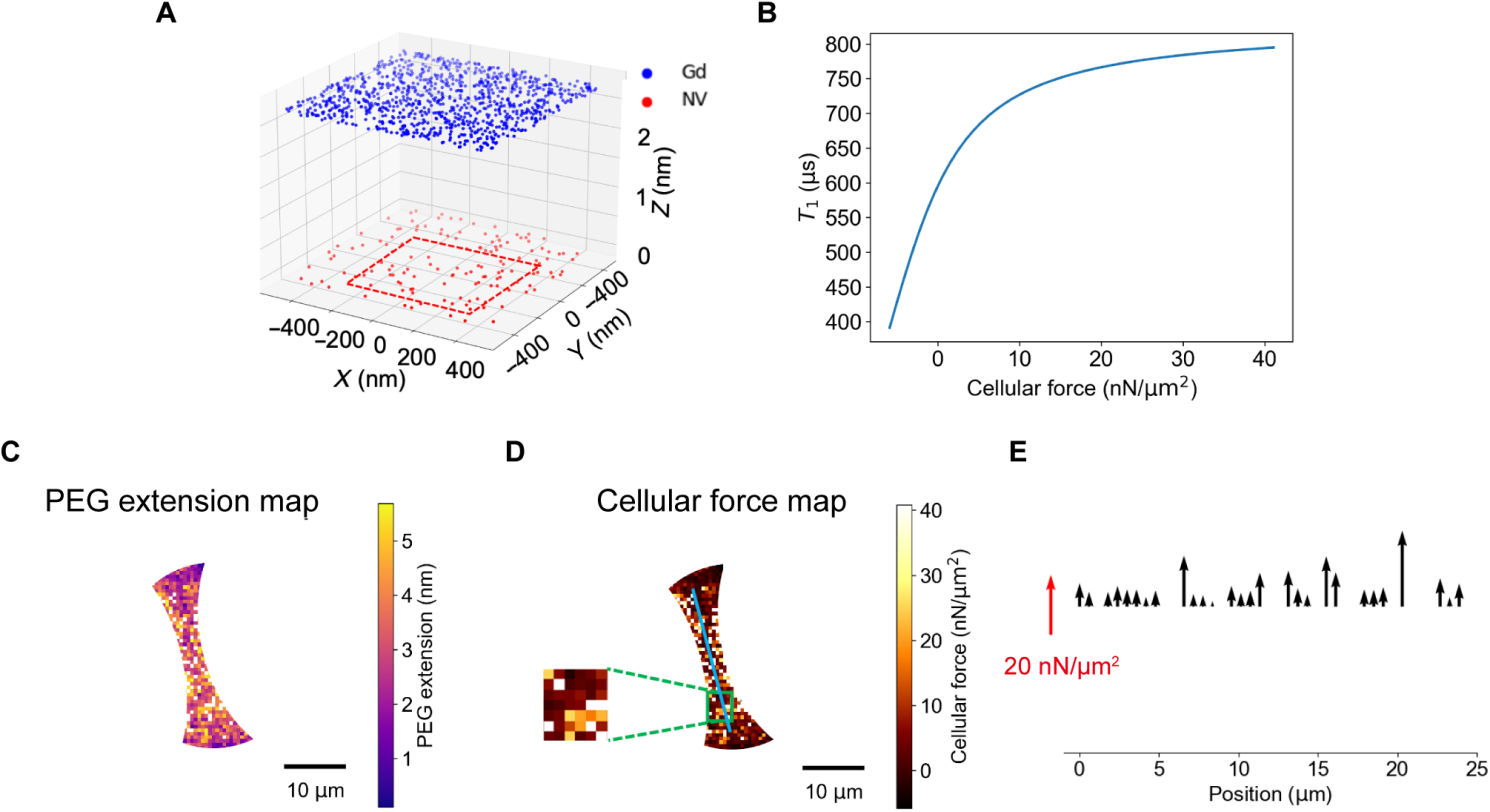
Simulation-assisted extraction of cellular forces from *T*_1_ mapping. (**A**) The schematics show the simplified model: A layer of NV centers is randomly distributed underneath the upper surface of a diamond membrane with a given depth and fixed density; a layer of the Gd^3+^ complexes attached to the PEG acts as a randomly fluctuating spin bath; the aforementioned two layers are separated by spring-like PEG molecules and their force-induced extension is described by worm-like chain model. The red rectangular area represents the minimum sensing area adopted (600 nm by 600 nm). (**B**) The simulated relationship between cellular forces and *T*_1_ value. The extracted (**C**) PEG extension map, and (**D**) cellular traction force map of the cell body in one chosen cell (the same one in Fig. 6B). The inset is the enlarged view of the selected rectangular area. (**E**) Force profile along the blue line drawn in (D). The direction of the cellular force exerted on the PEG polymer is normal to the diamond surface.

The force exerted on the QDMTM can be further quantified, as the cell adhesive forces could be revealed by relative *T*_1_ changes (Fig. 6). On the basis of the model built above for measuring the cellular force (Fig. 7A), the distance between Gd^3+^ molecules and NV centers plays a key role in determining *T*_1_ values, and the quantitative relationship between them can be derived from Monte Carlo numerical simulation (more details shown in Materials and Methods). Meanwhile, PEG extending and collapse under cellular forces is unregulated, but the vertical distance between Gd and NV has the most substantial effect on *T*_1_, so here we consider the change of PEG length in the vertical direction. By combining the extended WLC model, the relationships between cellular traction force and *T*_1_ can be obtained as shown in Fig. 7B. According to the relationships, we have reconstructed the cellular force of the cell exerted on the PEG molecules, and the *T*_1_ map can be converted into PEG extension map (Fig. 7C) and cellular force map (Fig. 7D). Note that the effective measurement range of cellular force is corresponding to the *T*_1_ values between 391 and 795 μs (Fig. 7B). The *T*_1_ reference value of 410 μs was obtained according to Fig. 4E. Thus, *T*_1_ below the reference value is considered a force-responsive polymer being compressed by the cell and conversely extended. However, only the extension of the polymer fits the WLC model, and the compression of the polymer can subsequently be investigated with a suitable model.

PEG length is an essential parameter both in experiments and simulations. PEG length influences the force-extension curve during the extended process. They determine the sensitivity and dynamic range of the sensor. In our experiment, the PEG [Molecular weight: ~1000 g/mol] full contour length is 6.20 to 7.98 nm. It is more sensitive to the force range from several piconewtons to around 30 pN (more details shown in Supplementary Materials simulation). On the basis of the simulation result, extensions of PEG range from 3.5 to 5.5 nm, and the tension loaded on a single PEG is around 10 pN, which agrees with the previous study (*19*, *70*). This semiquantitative model can guide choosing a suitable PEG length to tune the “most sensitive range” of our sensor, making it competent for sensing different force ranges, and enlarging the application scenarios of this development kit.

## DISCUSSION

In contrast to traditional optical methods using diverse fluorophores with a stochastic nature (*71*–*73*), our strategy is to use robust atomic defects in diamond, i.e., NV centers. This is achieved by coupling mechanical signals to the spin states of diamond defects through our designed force-responsive polymers (Fig. 1). It must be pointed out that the unprecedented sensitivity and precision of the proposed QDMTM are inherently guaranteed by the quantum nature of electronic spins of NV centers. This in conjunction with the fact that such an approach is totally fluorophores label-free can, in principle, overcome various difficulties, such as photobleaching, limited sensitivity, and ambiguities in data interpretation. Meanwhile, we believe that our method could serve as a good complement to the existing toolbox of TFM cellular forces measurements, as this method is intended to measure shear forces (being parallel to the substrate).

The presented QDMTM successfully distinguishes the various mechanical states of the adhered cells, including the well-spread cell edges, the bridged cell edges, as well as the cell-cell contact region. The results of cell force in different regions are in line with the presented knowledge that the integrin adhesive force is concentrated in the cell-polarized area and weakened by cell-cell interaction (*66*, *68*). These applications indicate the effectiveness and precision of QDMTM for cell force measurement. The current method can be, in principle, extended to nanoscale diamond particles, facilitating arbitrary cellular forces sensing as expected.

We have to admit that there is still room for improving QDMTM: (i) The stability of the optical system could be improved by introducing a fast autofocusing module (*74*) as any sample drift might affect the *T*_1_ measurements. (ii) It is doable to adopt MW-free (*75*) and/or fixed-tau (*43*) schemes for speeding up the *T*_1_ measurements. (iii) The pressure of the cell bodies exerted onto the sensors can be calculated according to a suitable model, which may be helpful in understanding the cell membrane tension.

Overall, this force-sensing tool, namely, the QDMTM, will allow enhanced sensitivity as well as resolution in time and space in comparison to available TFM. Also, quantum tension sensors may be reused after cleaning which will also enhance the absolute precision of sensors for comparing different samples. It can fundamentally change the way how we study important issues like cell-cell or cell-material interactions and hence bring impact to the field of biophysics and biomedical engineering. In addition, the data on the cellular forces transmitted through cell adhesions to be generated by this study are also expected to be useful in guiding and assisting the development of future theories on mechanosensing and mechanotransduction.

## MATERIALS AND METHODS

### Materials

(3-Isocyanatopropyl)trimethoxysilane (MeO)_3_-Si-NCO), triethylamine, tetraethyl orthosilicate (TEOS), 1,2-bis(triethoxysilyl)ethane (BTSE), GdCl_3_·6H_2_O, EDTA disodium salt (EDTA-2Na), (1R,8S,9s)-Bicyclo[6.1.0]non-4-yn-9-ylmethyl N-succinimidyl carbonate (BCN-NHS) were purchased from Sigma-Aldrich (Shanghai, China). 2,2′,2″-(10-(1-Carboxy-4-((2-(2,5-dioxo-2,5-dihydro-1H-pyrrol-1-yl)ethyl)amino)-4-oxobutyl)-1,4,7,10 tetraazacyclododecane-1,4,7-triyl) triacetic acid (maleimide-DOTA-GA) were purchased from Chematech (France). The peptide NH_2_-cyclo(-Arg-Gly-Asp-D-Phe-Lys)-SH (CycloRGDfK) was synthesized by GL Biochem Ltd. (Shanghai, China). N_3_-PEG-NH_2_ (Molecular weight: ~1000 g/mol) was synthesized by JenKem Technology (Beijing, China). All organic solvents are purchased from Acros (Germany) unless otherwise stated. The single crystalline diamond plates (2 mm × 2 mm × 0.03 mm, Applied Diamond Inc., Electronic Grade) were used to force the sensor diamond by implanting ^15^N^+^ ions into the diamond with 5 keV per atom. The implanted nitrogen atoms have a mean depth of 5 ± 2 nm and are then annealed in a vacuum tube furnace to 800°C to form NV centers.

### Construction of sensing platform based on diamond membrane

Detailed information on the synthetic force-responsive polymers and constructed diamond force sensor are provided in the Supplementary Materials (fig. S4). Briefly, the single crystalline diamond plates (2 mm × 2 mm × 0.03 mm, Applied Diamond Inc., Electronic Grade; 3 mm × 3 mm × 0.25 mm, Element Six, Optical Grade) were chemically activated using freshly prepared piranha solution. A thin layer of hybrid silica was then covalently modified onto the pristine oxygen-terminated diamond surface. Subsequently, Silane-PEG-N_3_ was grafted onto the silanization diamond surface, followed with modification with BRD molecules by the SPAAC reaction. Last, Gd^3+^ ions were loaded, and force-sensing platform can be established. To further regenerate the surface and promote recyclability, the force-responsive polymer modified diamond is easily cleaned by NaOH solutions and piranha washes. In addition, the synthesis of BCN-RGD-DOTA molecule required two steps: first, CycloRGDfK reacted with Maleimide-DOTA-GA by click reaction; second, condensation of BCN-NHS reacted with the product of RGD-DOTA (fig. S1). Last, it is modified to the surface by the SPAAC reaction of the azide group on PEG and Gd^3+^ ions were loaded by chelation.

### Characterization of constructed sensing platform

 Constructing a quantum sensing platform is essential for cellular forces measurements. Characterizing its elemental composition, morphology, ligand density, and functional surface thickness can be achieved using techniques like XPS, AFM, QCM, and ellipsometry, correspondingly. As it was difficult to evaluate the thin layer coating on the diamond surface via standard ellipsometry, the polymer immobilization was analyzed on a silicon model deposited with the silica layer (purchased from Ted Pella, Inc., 5 × 5 mm diced silicon wafer). Meanwhile, the silica QCM chips were used for the QCM test. Detailed information on the test parameters has been provided in the Supplementary Materials.

### Widefield quantum diamond microscopy

This widefield quantum diamond microscope mainly includes three subsystems—the optical system, the MW system, and the control system. The optical system, based on a customized widefield fluorescence microscope, provides efficient optical initialization and readout of NV centers in a large field of view. The 532-nm laser (Changchun New Industries, MGL-FN-532-1 W) passing through an acousto-optic modulator (Gooch & Hoosego, 3250-220) is focused on the back-focal plane of a 100× oil objective with 1.50 NA (Olympus, UAPON100XOTIRF). The NV fluorescence is filtered with a long-pass filter (Thorlabs, FELH0650) and imaged on a 512 × 512 air-cooled EMCCD (Teledyne Photometrics, Evolve 512 Delta) with an effective pixel size of 96 nm under the focal length of an imaging lens at 300 mm (Thorlabs). A 561-nm long-pass dichroic mirror (Semrock, Di03-R561-t1-25 × 36) is for reflecting the excitation beam into the objective and collecting the fluorescence longer than 561 nm. In the MW system, the microwave generated by a microwave source (ROHDE&SCHWARZ, SMBV100A) passing through a MW switch (Mini-Circuits, ZASWA-2-50DRA+) is amplified by a MW amplifier (Mini-Circuits, ZHL-16 W-43-S+) and exerted on the NV centers by a customized Omega-shaped MW antenna, then transmitted to the terminator. The control system is made up of a computer and a pulse streamer (Swabian Instruments, Pulse Streamer 8/2) for the data transfer and synchronizes the entire system.

### NV spin relaxometry measurements

After being polarized by a green laser pulse, the electron spin of the NV centers will relax to a thermal equilibrium state from the polarized state which is named longitudinal relaxation. The longitudinal relaxation rate Γ_1_ is principally dominated by spin-lattice interaction and fluctuating magnetic field generated by the nearby spin impurities, such as ferritin and Gd^3+^ used in the experiments to modulate the relaxation rate.

In our sensing protocols, the NV center is polarized to ∣0⟩ state by a 1−μs laser pulse with the power of 18 kW/cm^2^ first. After 500 ns, a π pulse of microwave is exerted to flip the NV center from ∣0⟩ state to ∣1⟩ state. Then, after a waiting time τ, another 1−μs laser pulse is applied to read out the spin state of the NV center and polarize the NV center. The above pulse sequences as a unit sequence will be repeated tens of thousands of times to obtain a bright enough fluorescent signal. Because of the camera’s inability to switch on and off quickly, the camera remains in an exposure state throughout the tens of thousands of repetitions of the unit sequence with a specific waiting time τ. To reduce the influence of the background signal, we repeated the above unit sequence but turned off the microwave to perform a control measurement. Therefore, the NV center relaxed from the ∣0⟩ state to the ∣1⟩ state in this measurement. By performing element-wise division between the image matrices without and with microwave, the impact of the background signal will be minimized and the *T*_1_ trace of the fluorescence intensity could be obtained. Last, through fitting the *T*_1_ fluorescence trace with the formula of $I\left(\tau \right)=a\cdot e^{-{\left(\frac{\tau }{T}\right)}^{b}}+c$ , we can get the *T*_1_ value.

### Seeding cells on the diamond-based sensing platform

According to the cell force measurement, we immobilized force sensor diamond (2 mm × 2 mm × 0.03 mm, Applied Diamond Inc., Electronic Grade) onto microwave antennas with an omega structure (280 μm in diameter) by using polydimethylsiloxane cured at 60°C (Dow Corning Sylgard 184; monomer: cross-linker = 10: 1). Samples were sterilized for 1 hour with 70% ethanol. The density of 10^4^/ml NIH 3T3 cells was cultured on the above-encapsulated diamond slides for 6 h and stability of cell adhesion was observed.

### Measurements of cell adhesion forces via NV spin relaxometry

Cells were washed once with cell culture medium and twice with PBS before fixation with 4% paraformaldehyde at room temperature for 15 min. Force sensor samples with adherent mature cells were then washed three times with PBS. Thoroughly cleaned diamonds were immersed in PBS for *T*_1_ testing at room temperature. For Δ*T*_1_ of Fig. 6D obtained by selecting the region *T*_1_ (Fig. 6, A to C, marked i, ii, iii, and iv) minus the reference value (obtained by Fig. 4E).

### Theoretical model for quantifying the relationship between cell forces and *T*_1_ value

A numerical simulation model is built to simulate the longitudinal relaxation time of the NV center under the magnetic disturbance of the Gd^3+^ ions (for more information about the numerical model please, refer to Supplementary Materials). In this model, the location of the NV centers is randomly generated with an orientation randomly selected from four given directions of $\left[\bar{1}11\right]$ , $\left[1\bar{1}1\right]$ , $\left[11\bar{1}\right]$ , and $\left[\bar{1}\bar{1}\bar{1}\right]$ by the Monte Carlo simulation (the bulk diamond is [100] cut). The density and depth of the NV centers are 1000/μm^2^ and 5 nm (under the bulk diamond surface), respectively. The density of the Gd^3+^ is set to 9000/μm^2^ in simulation. The location of the Gd^3+^ in the *XY* plane is also generated randomly by Monte Carlo simulation.

For calculating the *T*_1_ of a single NV center, we only take the Gd^3+^ ions (with the same height) inside a circle with a diameter of 100 nm over the NV centers into consideration because the NV centers outside the circle have no interactions with the NV centers. Then, we recover the *T*_1_ fluorescence curve of the single NV centers based on the *T*_1_ value obtained in the simulation. Sum all the *T*_1_ fluorescence curves of the NV centers within the red dashed square of 600 x 600 nm^2^ (Fig. 7A) which corresponds to the effective pixel size on the sample plane under our setup configuration. Last, the *T*_1_ value of the NV centers under the interaction of the Gd^3+^ ions could be obtained by fitting the summed *T*_1_ fluorescence curve with a formula of $I\left(t\right)=A\cdot e^{-{\left(\frac{t}{T}\right)}^{b}}+c$ . By scanning the height of the Gd^3+^ ions from 0.3 to 6 nm in the numerical model, a relationship between the height of Gd^3+^ ions and the *T*_1_ values could be given, where the height of Gd^3+^ ions represents the length of the PEG polymers. Last, combining the WLC model (refer to Supplementary Materials for details) where the relationship of the force exerted on the PEG and extension of the PEG could be described, the relationship of the *T*_1_ value and the force exerted on the PEG could be given.

## Supplementary Materials

### This PDF file includes:

Supplementary Text Figs. S1 to S18 Table S1 References
