# Supplementary material for: Quantum-enhanced diamond molecular tension microscopy for quantifying cellular forces
Source: Sci Adv. 2024 Jan 24;10(4):eadi5300. doi: 10.1126/sciadv.adi5300 (PMC10807811; doi:10.1126/sciadv.adi5300)
Supplement: Supplementary file 1 — Supplementary Text Figs. S1 to S18 Table S1 References [file sciadv.adi5300_sm.pdf]

Science Advances
AAAS

Supplementary Materials for

Quantum-enhanced diamond molecular tension microscopy for quantifying cellular forces

Feng Xu *et al.*

Corresponding author: Zhiqin Chu, zqchu@eee.hku.hk; Qiang Wei, wei@scu.edu.cn

Sci. Adv. **10**, eadi5300 (2024)
DOI: 10.1126/sciadv.adi5300

This PDF file includes:

Supplementary Text
Figs. S1 to S18
Table S1
References

Synthesis of BRD (BCN-RGD-DOTA)

Synthesis of BRD was carried out in two-step reaction with high conversion rate, as shown in fig. S1. The 3.0 ml of dry dimethylformamide (DMF) was taken and added into 10 ml flask under argon flux to dissolve cyclo(-Arg-Gly-Asp-D-Phe-Lys) (CycloRGDfK, 3 mg, 0.004 mmol, Mw: 706.32 g/mol). Additional 1.0 ml DMF solution was taken to dissolve 2,2',2"-(10-(1-carboxy-4-((2-(2,5-dioxo-2,5-dihydro-1H-pyrrol-1-yl)ethyl)amino)-4-oxobutyl)-1,4,7,10 tetraazacyclododecane-1,4,7-triyl) triacetic acid (Maleimide-DOTA-GA, 3.1 mg, 0.0052 mmol, Mw: 598.26 g/mol, 1.25 eqv. to thiol groups of CycloRGDfK). The Maleimide-DOTA-GA solution was slowly added into the CycloRGDfK solution by dropwise. The mixture was stirred at room temperature for 24 hours. Further purification was not required, and it was directly used in the next step. The m/z of RGD-DOTA was detected by LC-MS. M^+: m/z 1304.58; found m/z 1305.6 (M^+ + H); m/z 435.9 (M^+ + 3H)/3; m/z 653.3 (M^+ + 3H)/3. The CycloRGDfK peak was undetected, demonstrating the high reaction efficiency.

Next, the (1R,8S,9s)-Bicyclo[6.1.0]non-4-yn-9-ylmethyl N-succinimidyl carbonate (BCN-NHS, 0.88 mg, 0.003mmol, 0.8 eqv to amine of RGD-DOTA) was dissolved in 1.0 ml of DMF solution and added to the obtained RGD-DOTA mixture, followed by stirring at room temperature for 24 hours. After the reaction, the BCN-RGD-DOTA solution was obtained. The m/z of the products were analyzed by MADTI-TOF-MS. M^+: m/z 1480.67; found m/z 1481.1066 (M^+ + H). The RGD-DOTA and BCN-RGD-DOTA peaks were detected, indicating successful synthesis of BCN-RGD-DOTA.

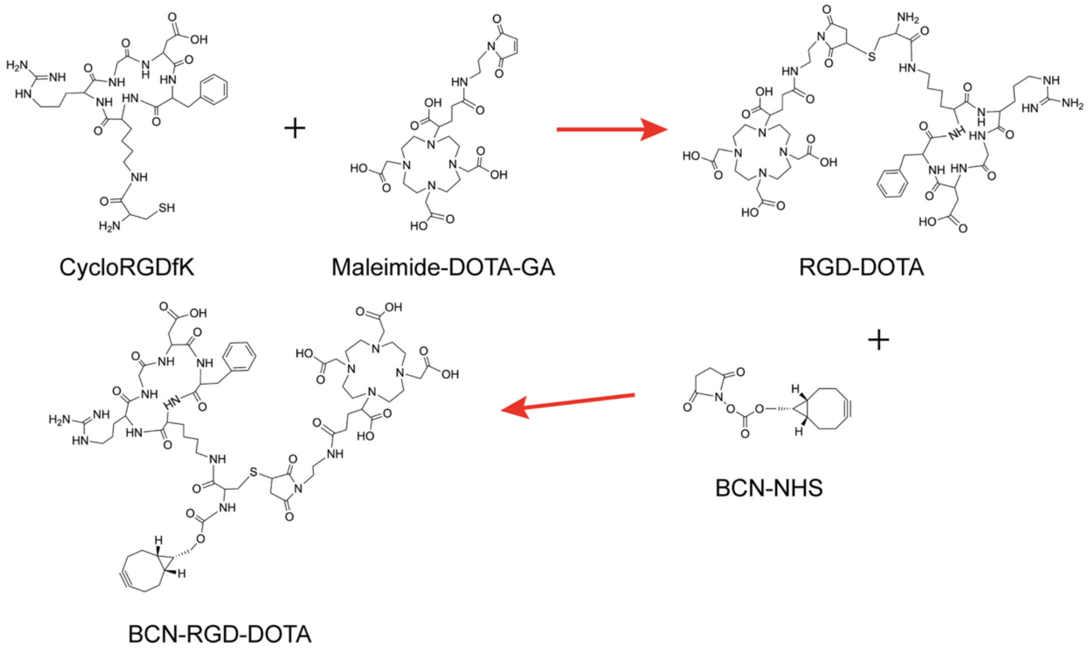

Fig. S1.

General procedure for the synthesis of the BCN-RGD-DOTA (BRD).

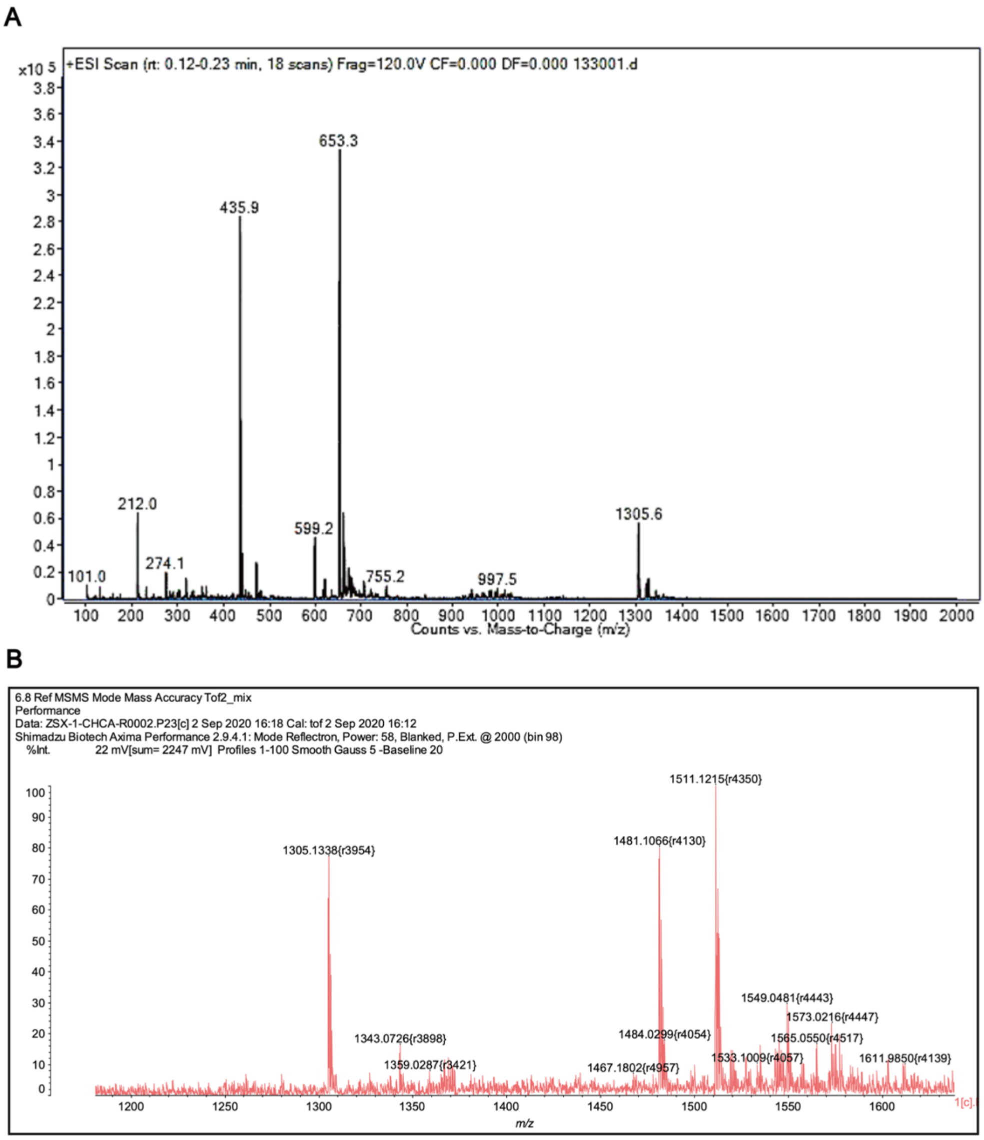

Fig. S2.

Mass spectrometry for measurement of molecular mass. (A) LC-MS spectrum of the crude product of RGD-DOTA. **(B)** MADTI-TOF-MS spectrum of the crude product of BCN-RGD-DOTA (BRD).

Synthesis of Silane-PEG-N_3

The 1 ml toluene was added into the flask under argon flux to dissolve N_3-PEG-NH_2 (10.0 mg, 0.01 mmol, Mw: ~1000 g/mol). The 4.0 µl of (3-isocyanatopropyl)trimethoxysilane ($(MeO)_3$-Si-NCO, 2 eqv. to amino groups) and 1.5 µl of triethylamine (TEA) were added into reaction flask under argon flux as well. The argon flux was maintained for 30 min, and then the reaction solution was kept stirred at 60°C for 24 hours. The products were purified by precipitation from n-hexane to obtain a viscous yellowish liquid (Silane-PEG-N_3). ^{1}H NMR (DMSO-d6), δ ppm: 0.4-0.6 (Si-C**H**$_2$, 2H), 1.1-1.2 (NH-CH_2-C**H**$_2$-CH_2, 2H), 2.8-3.0 (N_3-C**H**$_2$, 2H), 3.0-3.2 (N_3-CH_2-C**H**$_2$, NH-C**H**$_2$-CH_2, 4H), 3.45-3.65 (PEG backbone, O-C**H**$_3$, C**H**$_2$-C**H**$_2$-NH).

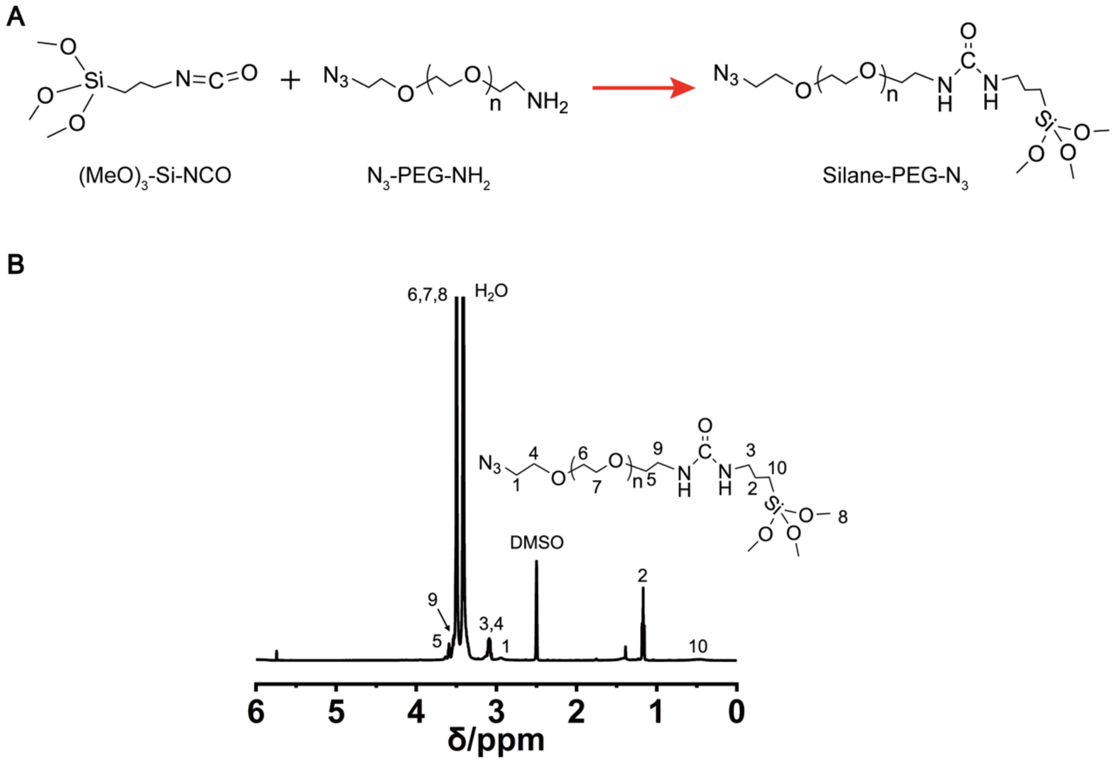

Fig. S3.

Synthesis of Silane-PEG-N_3. (A) General procedure for the synthesis of the Silane-PEG-N_3. **(B)** ^{1}H NMR spectrum of Silane-PEG-N_3 in DMSO-d6.

Constructing force-responsive polymers on diamond surface

Single-crystalline diamond slides were sonicated in acetone and isopropanol for 5 min in each and dried with nitrogen. The diamond slides were cleaned and chemically activated by freshly prepared piranha solution (H_2SO_4/H_2O_2=7:3) at 90°C for 1 hour, followed by thoroughly rinsing with ultrapure water and ethanol as well as drying with nitrogen (the samples were named as Pristine Diamond).

The 20 µl tetraethyl orthosilicate (TEOS) was added to a mixture of ethanol (2850 µl), ultrapure water (150 µl) and hydrochloric acid (10 µl) by dropwise for 1 hour. Then 10 µl of 1,2-bis(trimethylsiloxyl)ethane (BTSE) was added by dropwise as well, and the hydrolysis reaction was continued for another 1 hour. Afterwards, the cleaned Pristine Diamond was placed into this solution for 6 hours. After the reaction, the diamond slides were cleaned with ethanol and dried with nitrogen, obtaining hybrid silica-modified diamond surfaces (the samples were named as Silica-coated).

Then, the Silica-coated diamond slides were placed in a flask and immersed in a solution of 10 mg/ml Silane-PEG-N_3 dissolved in toluene. The reaction solution was kept at 55°C for 24 hours. After the reaction, the cleaned slides were kept under vacuum at 80°C for 1 hour and in an incubator at 60°C overnight. Subsequently, it was immersed in an anhydrous ethanol solution for 12 hours to quench the unreacted isocyanate groups on the slide surfaces and obtained PEGylated diamond surfaces (the samples were named as PEGylated)

The PEGylated surfaces were immersed in 1.0 ml of the BRD solution for 24 hours. Afterwards, the slides were removed and washed with dimethylformamide and ethanol, followed by drying with nitrogen to obtain biofunctionalized diamond surfaces. Finally, the gadolinium ions were loaded by immersing the BRD-modified diamond slides into 0.5 mg/ml $GdCl_3 \cdot 6H_2O$ water solution for 2 hours and washed with ethylenediaminetetraacetic acid disodium salt (EDTA-2Na, 0.4 mg/ml) for 1 hour. after the chelation, the slides were cleaned with water and ethanol, and dried with nitrogen to achieve force-responsive polymers modified diamond surfaces (the samples were

named as Force sensor).

Silicon wafers were used for similar modification processes, named Si, Si-Silica-coated, Si-PEGylated, and Si-Force sensor.

Reuse of the diamond slides

The Force-sensor can be simply removed by NaOH and piranha solution, providing an easily recyclable NV quantum sensor. Briefly, the functionalized diamond slides were firstly immersed in 1 M NaOH solution at 80°C for 12 hours, and then in piranha at 90°C for 1 hour. The corroded slides were extensively rinsed with ultrapure water and sonicated with acetone and 2-isopropanol for 5 min in each and dried with nitrogen. Besides, the slides can be also soaked in a 1:1:1 mixture of nitric acid, perchloric acid and sulphuric acid at boiling temperature (*76*).

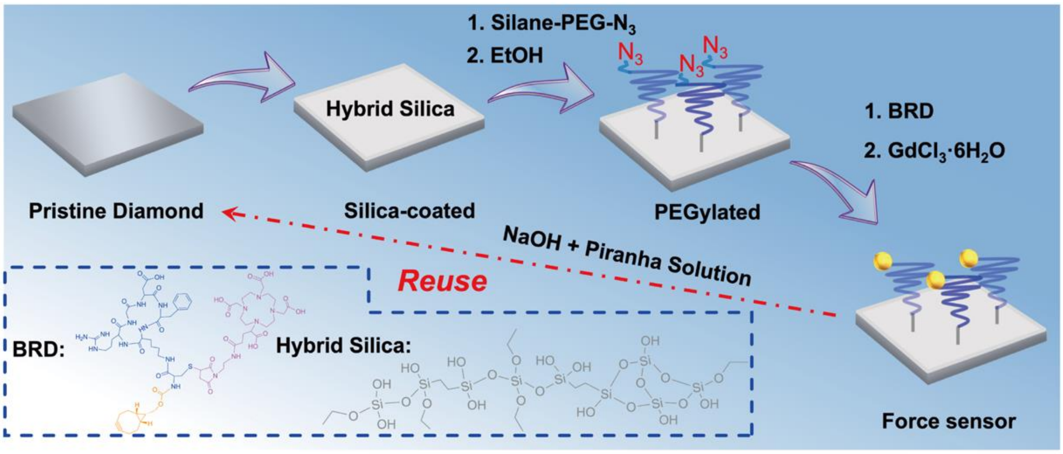

Fig. S4.

Schematic illustration of the functionalization process. The ultrathin hybrid silica layer was fabricated by BTSE and TEOS. The cell adhesive molecule (BCN-RGD-DOTA), consisting of an adhesion peptide (CycloRGDfK, blue in BRD) and a chelator (DOTA, pink in BRD), is immobilized to the azide terminal of the PEG polymer by the BCN-based (orange in BRD) SPAAC reaction.

Surface characterization

At each step of the functionalization procedure, we characterized the elemental composition, morphology and thickness of the functional surfaces by XPS, AFM, QCM and ellipsometry. Silicon wafer is utilized as model surface, instead of diamond slides, because it adapts to various surface characterization techniques such as ellipsometry, QCM and XPS (*77*).

X-ray photoelectron (XPS) spectroscopy

The surface elemental tests were carried out on silicon wafers with different coating steps by XPS (K-Alpha XPS, Thermo Scientific). The XPS measurements were performed on a Kratos system with 4×10^{-10} mbar base pressure, sample neutralization applying low energy electrons, hybrid mode, take off angle of electrons (0°), pass energy (160 eV), and excitation of photoelectrons by monochromatic $Al_{k\alpha}$ radiation (hν = 1486.6 eV) at 300W (15 kV Å~ 20 mA). The detected region was elliptically shaped (300 µm × 700 µm for main axes).

XPS analysis was used to quantitatively determine the chemical composition of the surface of the silicon wafers modified with hybrid silica and PEG. Compared to the unmodified Si, the intensity of the C-O (286.8 eV) and Si-O (103.6 eV) peaks on the Si-PEGylated and Si-Silica-coated surfaces were obviously increased (fig. S5B), wherein C-O and Si-O were affiliated to the backbone of PEG and silane, respectively.

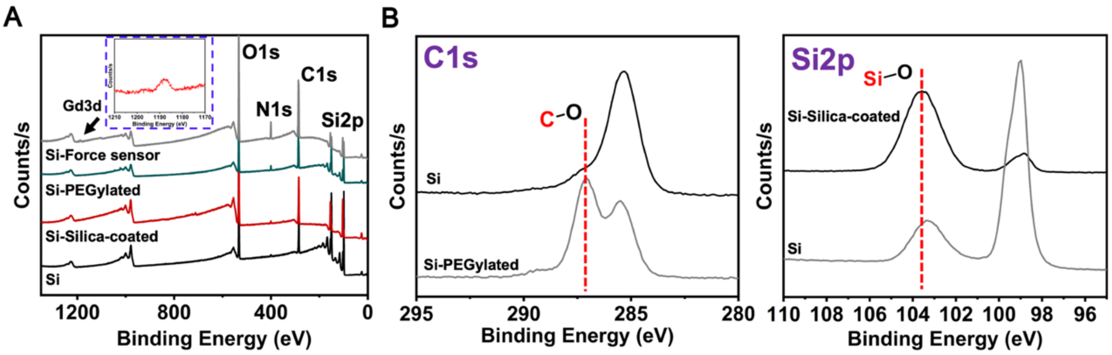

Fig. S5.

XPS analysis of functionalized surface elements. (A) XPS survey spectrum of C1s, N1s, O1s and Si2p signals after each functionalization step (insert represents Gd3d signal spectrum). **(B)** High resolution spectra of C1s and Si2p spectrum of Si, Si-Silica-coated and Si-PEGylated

Table S1 showed the relevant atomic compositions of different functionalized surfaces. After silica deposition, the content of O changed notably. The PEG grafting increased the N signal from 1.86 to 2.99% and the C/Si ratio was shifted to 1.43. After immobilizing the BRD and loading Gd^{3+}, the N signal further increased from 2.99 to 6.2%, the Gd signal appeared, and the C/Si ratio was shifted to 2.91, confirming the presence of BRD and Gd^{3+}.

In addition, after the chelation, EDTA-2Na was used to clean the free Gd^{3+} trapping in the PEG chains. The stability constants of EDTA-2Na with Gd^{3+} ions were lower than that of DOTA, thus, the chelated Gd^{3+} was stable during purification (*78*).

Table S1.

XPS elemental surface composition of Si, Si-Silica-coated, Si-PEGylated, and Si-Force sensor.

Samples	Elements (%)					
	[Si]	[C]	[N]	[O]	[Gd]	C/Si
Si	42.95	23.42	1.8	31.83	/	0.55
Si-Silica-coated	24.8	29.54	1.86	43.8	/	1.19
Si-PEGylated	27.82	39.9	2.99	29.29	/	1.43
Si-Force sensor	16.44	47.89	6.2	29.65	0.11	2.91

Ellipsometry

Silicon wafers with different steps of coatings were tested by ellipsometry, which was performed in the spectrum range of 380 to 1050 nm at the incidence of 70°, with an ellipsometer (SENpro, SENTECH Instruments GmbH, Germany). Each data point resulted from an average of at least 3 measurements, and the obtained sensor grams were fitted with a four-layer model (Si, SiO_2, organic layer, and air) using the analysis software SpectraRay/3. The model layer of 'silicon VIS+NIR' was used as substrate with n = 3.817 and k = 0.01576. The thickness of the PEG and hybrid silica layers was measured without BRD decoration. The layers were set as Cauchy layer and assumed to be constant (T = 2.0 nm, N0 = 1.56, and N1 = 104.5). The layers were further fitted by the Cauchy model.

Stability test

For the coating stability test, we soaked the Si-PEGylated in PBS solution for 5 days and then rinsed them three times with ultrapure water before drying with nitrogen gas.

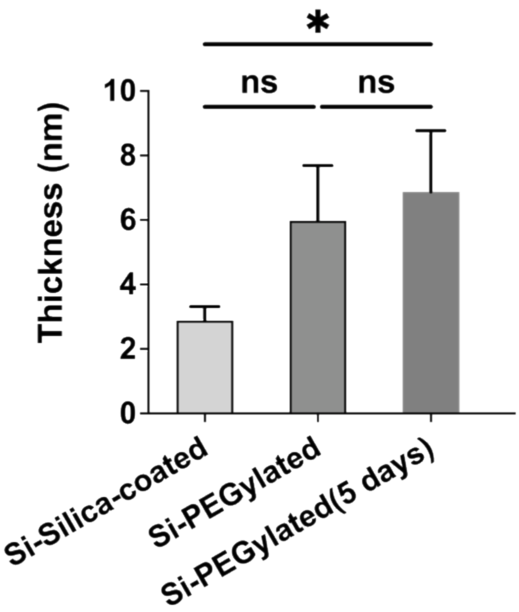

Fig. S6.

Ellipsometry for analysis of the thickness of the modification layer. The results showing the thickness of Si-Silica-coated and Si-PEGylated surfaces on silicon wafers before and after immersion in PBS solution for 5 days at room temperature (n=3, three technical replicates, p values were obtained by one-way ANOVA followed by Tukey's post hoc test, mean with standard deviation (S.D)).

Atomic force microscopy (AFM)

The surface morphology of the modified diamonds was recorded by NanoWizard 4 XP scanning probe microscopy (SPM) system (Bruker, USA) in the air and water under ambient conditions. The commercially available AFM Probe (TESP-V2) with a spring constant of ~ 37 N/m and resonance frequency of ~ 320 kHz was used in Tapping Mode, and the scanning rate was set at 0.8 Hz. SCANANSYST-AIR probes with a spring constant of 0.4N/m (Bruker, USA) were used in Quantitative Imaging (QI) Mode. The average surface roughness of the tested surfaces was analyzed by JPK Data Processing software and calculated from AFM images (1 × 1 μm^2 for functionalization for diamonds and 0.6 × 0.6 μm^2 for ferritin absorb on the PEGylated).

As confirmed by atomic force microscopy (AFM), we were able to deposit a uniform hybrid silica layer (Silica-coated: root mean roughness Rq = 332.1 pm) on the oxygen-terminated diamond surface (Pristine Diamond: Rq = 304.1 pm). The roughness slightly increased after PEG immobilization (PEGylated: Rq = 528.7 pm). The final surface roughness Rq = 739.6 pm can be obtained after the modification of force-responsive polymer (Force sensor).

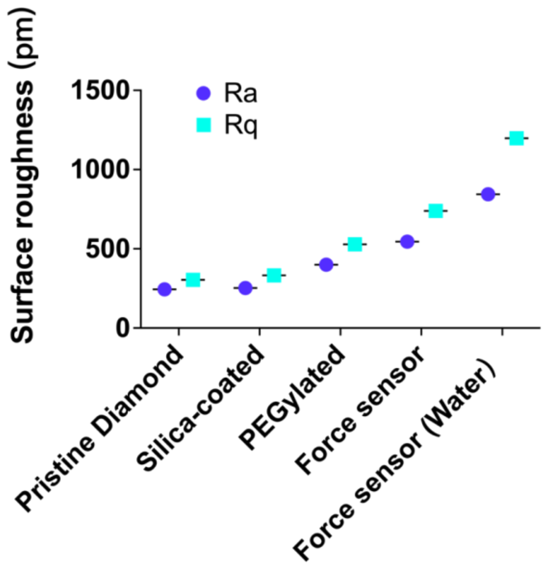

Fig. S7.

AFM characterization of the roughness of the functional diamond surfaces after each functionalization step.

Quartz crystal microbalance

Quartz crystal microbalance (QCM) with dissipation (Q-Sense E1, Sweden) was used to study the online coating of BRD and the BSA adsorption on the surfaces with different modifications. QCM with dissipation allows the monitoring of changes in resonance frequency (Δf) and dissipation (ΔD) of a piezoelectric quartz crystal as a function of time. The f and D were recorded at the fundamental frequency (4.95 MHz) and its 3rd, 5th, 7th, 9th, 11th, and 13th overtones. Only the 3rd overtone was shown in the sensor grams.

The whole measurement was performed at 25 °C. The Sauerbrey equation was used to calculate the mass of the adsorbates [$\Delta m = C \times \Delta f/3$, where Δm is the change in mass, C is the mass sensitivity constant of the quartz crystal ($-17.7\ ng \cdot cm^{-2} \cdot Hz^{-1}$), and Δf is the overtone-normalized frequency change] as the ΔD values were low.

The PEG-modified silica QCM chips (LOT-Quantum Design GmbH, Darmstadt, Germany) were fabricated via the protocol above. For monitoring the online coating of BRD molecular, the cleaned PEG-coated chips were inserted into flow chamber (QFM 401, QSense, Sweden, internal volume of 40 μl) and incubated in DMF/H_2O (1:9 v/v) with a flow rate of 0.1 ml/min. After baseline equilibration, a solution of BRD (Fig. S8a, 1 mg/ml in DMF/H_2O (1:9 v/v)) was pumped into the flow chamber at the same rate. After 1 hour of online incubation, the flow chamber was alternately rinsed with Milli-Q water, aqueous solution of deconex 1% (w/w, Borer Chemie AG, Switzerland), and Milli-Q water.

The protein adsorption was measured similarly. The coated sensors were inserted into the titanium flow chamber (QFM 401, Q-Sense, Sweden, internal volume of 40 μl) and incubated in PBS buffer. After baseline equilibration, PBS buffer was pumped into the flow chamber for 10 min, and then the protein solution 1 mg/mL BSA solution was pumped into the flow chamber, followed by washing with PBS. Fig. S8B shows the BSA absorption on the PEG and complete force-responsive polymers modified-silica QCM chip. The coatings were efficient enough to resist the protein.

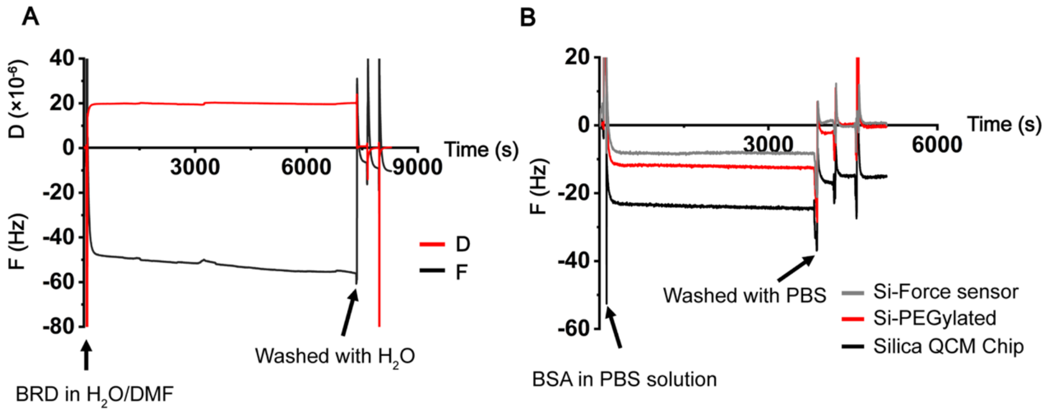

Fig. S8.

Investigating the surface properties with QCM. (A) QCM frequency (F) and dissipation (D) shift as a function of time during binding of BCN-RGD-DOTA (BRD) on PEG-coated gold QCM chip (contain hybrid silica layer) in DMF/H_2O (v/v: 1:9). **(B)** QCM frequency shift of the adsorption of bovine serum albumin (BSA) on silica QCM Chip, Si-PEGylated, Si-Force sensor.

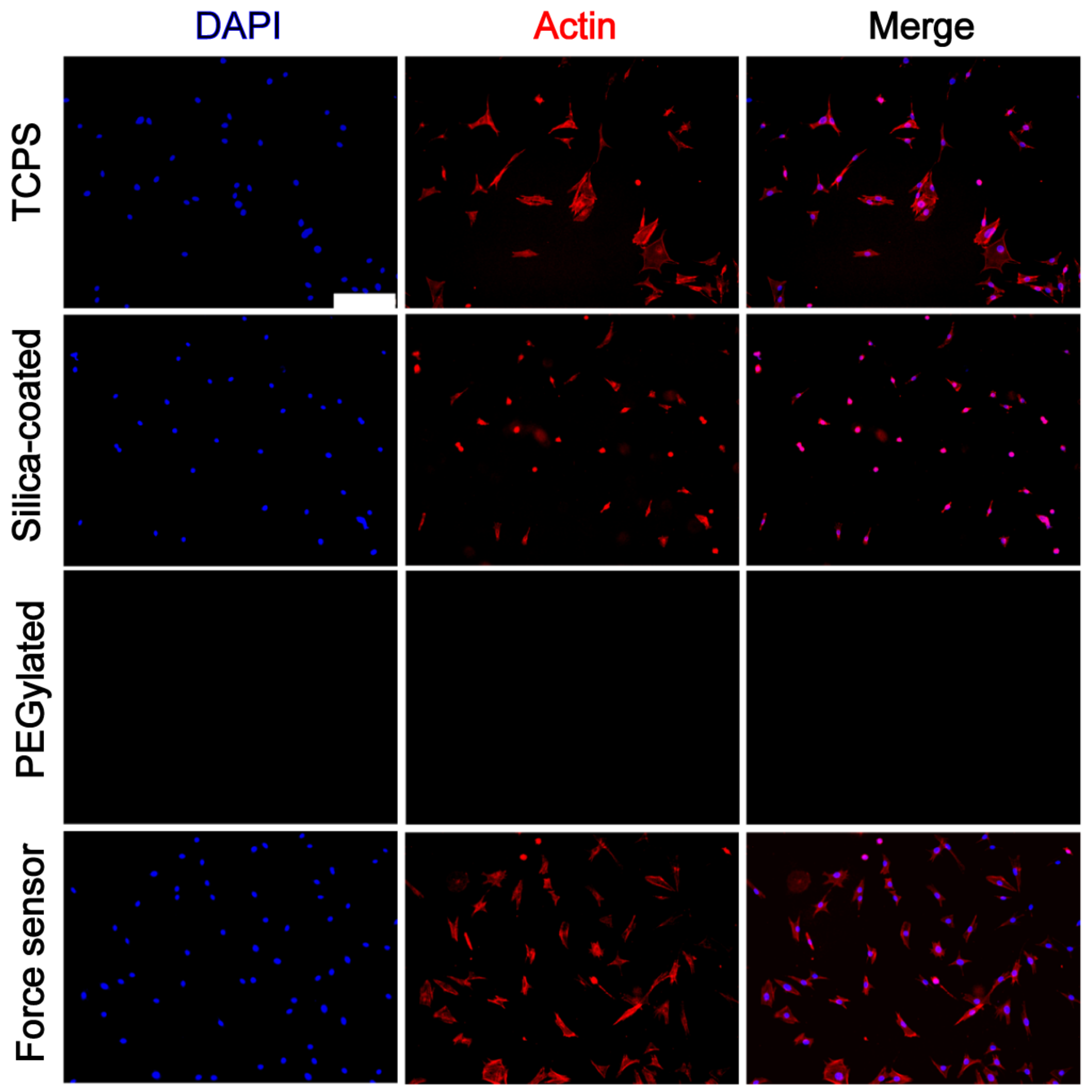

Fig. S9.

Representative fluorescence images of cell adhesion on different functionalized diamond surfaces. The original-sized images of NIH 3T3 stained with the cytoskeleton (Phalloidin, red) and nuclei (DAPI, blue) after culturing for 16 hours. The scale bar is 100 μm.

After 3 days of cell culture with NIH 3T3 fibroblasts, the tissue culture polystyrene (TCPS) and Silica-coated diamond surfaces were covered with well-spread cells. Whereas almost no cells adhered on PEGylated surfaces (Fig. S10).

Siloxane materials are reported to be not hydrothermally stable, herein, BTSE not only produces more Si-OH but also increases the stability of the silica layer (*79, 80*). Besides, the hybrid silica can be further modified without decreasing the sensitivity of the measurement. These data demonstrated that the introduction of an “active” hybrid silica layer on the diamond surface is imperative to stabilize the force-responsive polymers.

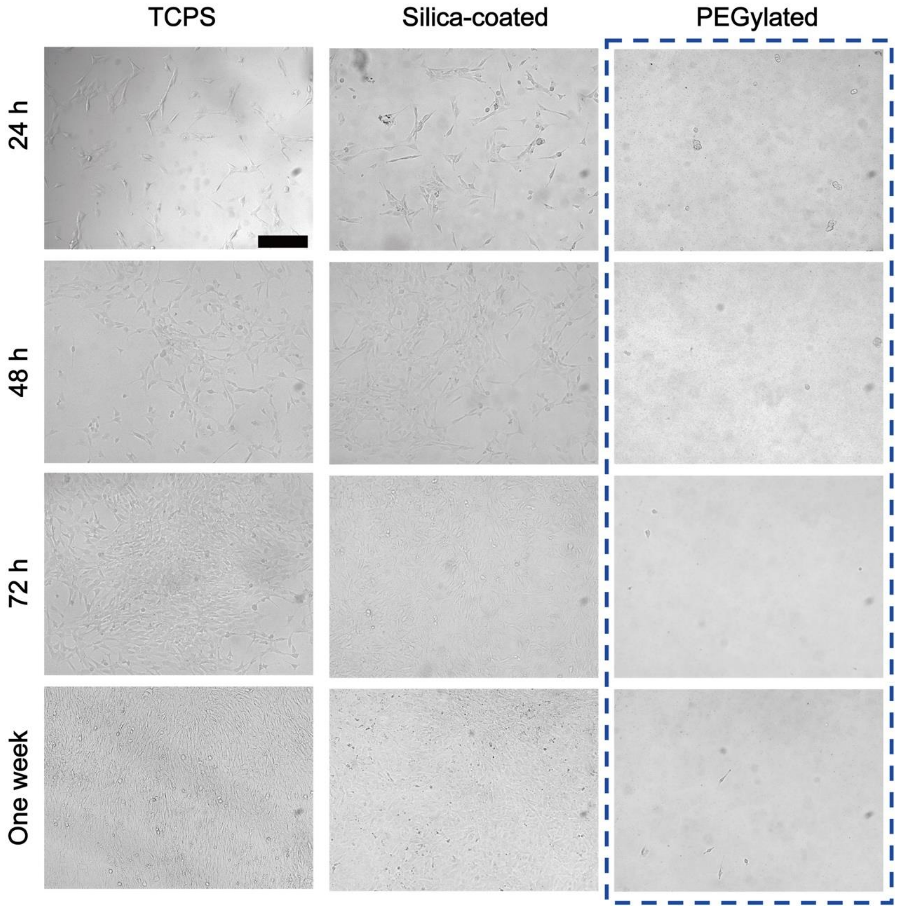

Fig. S10.

Cells grown on different substrates. Transmitted light images of NIH 3T3 cells adhered on the Silica-coated, PEGylated functional diamond surfaces after 1 day, 2 days, 3 days, and 7 days of cell culture (scale bar indicates 100 μm).

When the diamond slides were immersed in the 1 mM $GdCl_3$ solution, the T_1 decreased 13 times compared with it in the pure water. We blocked part of the diamond with polydimethylsiloxane (PDMS). The PDMS decreased the T_1 value of the slide to 68% in pure water, which may attribute to the impurity of the metal catalyst for PDMS synthesis (*81*). After immersing in the $GdCl_3$ solution, the T_1 value of the PDMS blocked region was 4 times higher than the unblocked region because the PDMS reduced the diffusion of Gd^{3+} to the diamond surface.

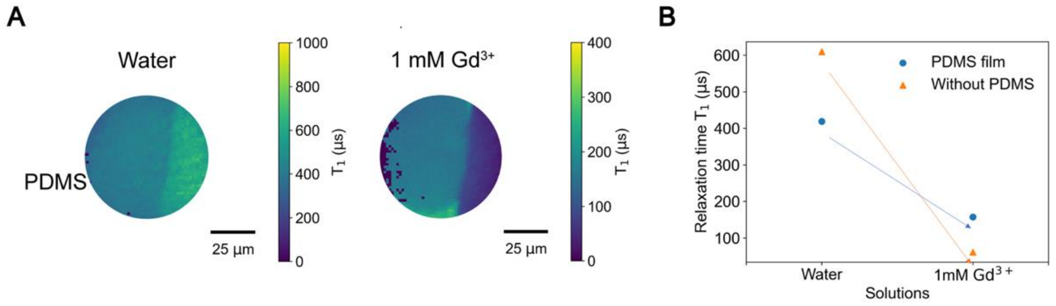

Fig. S11.

T_1 measurement of diamond membrane immersed in different solutions. (A) T_1 mapping of pure diamond membrane (without surface modifications) in the presence of ultrapure water and 1 mM Gd^{3+} solution, respectively, part of which was covered by PDMS (40X air objective). **(B)** Corresponding mean values of T_1 mapping in Fig. S11A.

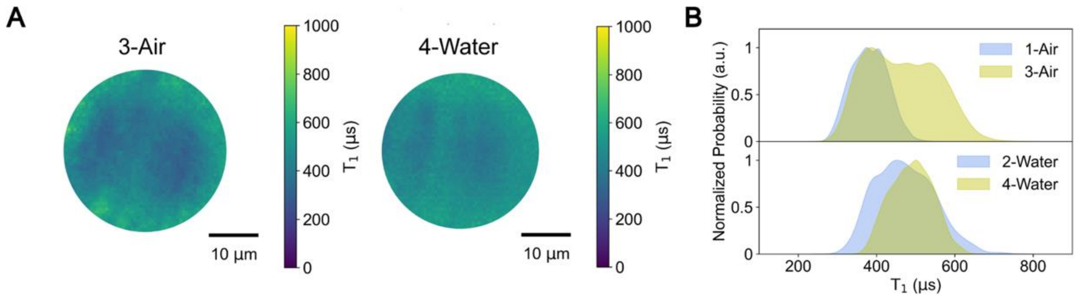

Fig. S12.

T_1 measurements of the diamond force sensor immersed in different solutions. (A) T_1 measurements of the force sensor in air and ultrapure water environments after another cycle, respectively. **(B)** The histogram of T_1 values within the T_1 mapping is shown in Fig. S12A. 1, 2, 3, 4 means that the T_1 measurements were performed successively in the same position in different environments, where 1, 3 corresponds to air conditions and 2, 4 to water conditions. Each time the diamond is in a different environment, it is necessary to wait for at least 12 hours for the molecular conformation to change sufficiently.

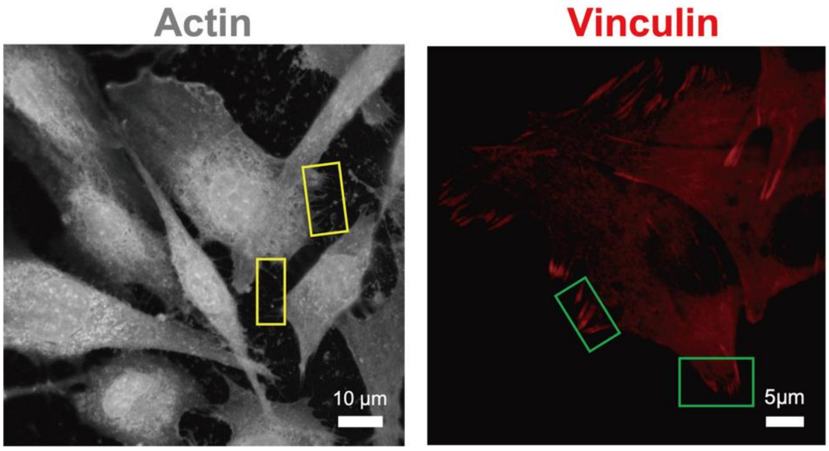

Fig. S13.

Representative fluorescence images of F-actin and vinculin of NIH 3T3 staining after T_1 measurements. Yellow boxes indicate pseudopodia and green boxes indicate focal adhesions, respectively. According to the images, the length of ~3 μm can be defined as the cell edge region (Fig. 6, A to C marked i, ii, iii, iv).

Cell culture

NIH 3T3 fibroblasts (ATCC) were cultured in standard DMEM (Gibco, 11965092) supplemented with 10% bovine growth serum (Gibco, 16030074) and 1% penicillin/streptomycin (Gibco, 15140122) at 37°C with 5% CO_2.

According to the stability test of the functional coatings, NIH 3T3 cells were seeded on the functionalized diamond slides (3 mm × 3 mm × 0.25 mm, Element Six, Optical Grade) for 16 hours to 7 days, followed by optical or fluorescent images acquisition.

Immunofluorescence staining and microscopy

Cells were washed once with cell culture medium and twice with PBS before fixation with 4% paraformaldehyde at room temperature for 15 min. Samples were then washed three times with PBS. Cells were permeabilized with 0.25% v/v Triton-X 100 in PBS for 10 min at room temperature, then washed three times with PBS. Nonspecific antibody adsorption was blocked by incubating samples with 1% w/v bovine serum albumin in PBST (0.1% v/v Triton-X 100 in PBS (PBST)) at room temperature for 45 min. Following primary antibody incubation (1:100, vinculin, Thermo), samples were washed twice with PBST and three times with PBS. Samples were then incubated with secondary antibodies, phalloidin 488 (Abcam, 1:1000) and DAPI at room temperature for 1 h, followed by washing three times with PBS. Immunofluorescence images were acquired and analyzed via confocal microscope (Zeiss710).

Statistic assay

Data of the cell adhesion study, measurement of T_1 and coating thickness are represented as mean ± standard deviation (S.D). Group differences were conducted by one-way ANOVA. P-values < 0.05 were considered statistically significant (*$p < 0.05$, **$p < 0.01$, ***$p < 0.001$, ****$p < 0.0001$). All statistical analyses were performed with GraphPad Prism 8.

Simulation

The physical model of calculating Gd's influence on the T_1 of NV Center

Starting from Fermi's Golden rule, the relaxation rate of NV center can be derived as follows (*82*):

$$\frac{1}{T_1} = \frac{1}{T_1^{bulk}} + 3\gamma_e^2 B_\perp^2 \frac{\tau_c}{1 + \omega_0^2 \tau_c^2}$$

where T_1^{bulk} is the T_1 of NV in the bulk diamond. (We set it as 900 ns in this simulation based on experiment data.), ω_0 is the NV zero-field splitting, where $\frac{\omega_0}{2\pi} = 2.87GHz$ (*83*).

γ_e is the electron gyromagnetic ratio.

$\tau_c = \frac{1}{R_{Gd,tot}}$, where $R_{Gd,tot} = R_{dip,Gd} + R_{vib} + R_{trans} + R_{rot}$.

$$\hbar R_{dip} = \sqrt{\sum_{i \neq j} \langle H_{ij}^2 \rangle} = \frac{\mu_0 \gamma_e^2 \sqrt{6} C_s}{4\pi} \left(\sum_{i \neq j} \frac{1}{r_{ij}^6} \right)^{\frac{1}{2}}$$

R_{trans} and R_{rot} are due to the Brownian motion. The vibration term R_{vib} originates from the electronic spin relaxation of Gd, and it has a fixed value at 298 K which is 2.1 GHz (*84*). R_{trans} is negligible.

$$R_{rot} = \frac{k_B T}{8\pi a^3 \eta f_r}, \; where \;\; f_r = \left(\frac{6a_s}{a} + \frac{1 + \frac{3a_s}{a + 2a_s}}{\left(1 + \frac{2a_s}{a}\right)^3} \right)^{-1}$$

a_s and a are the molecule radius of the solution and the Gd molecule. Here, we set $a_s = 0.14\,nm, \; a = 0.39\,nm.$

$$B_{\perp,i}^2 = \langle B_{x,i}^2 \rangle + \langle B_{y,i}^2 \rangle = Tr\{\rho(B_{x,i}^2 + B_{y,i}^2)\} = \left(\frac{\mu_0 \gamma_e \hbar}{4\pi}\right)^2 C_s \frac{2 + 3sin^2\alpha_i}{r_i^6}$$

r_i and α_i are shown in Fig. S14.

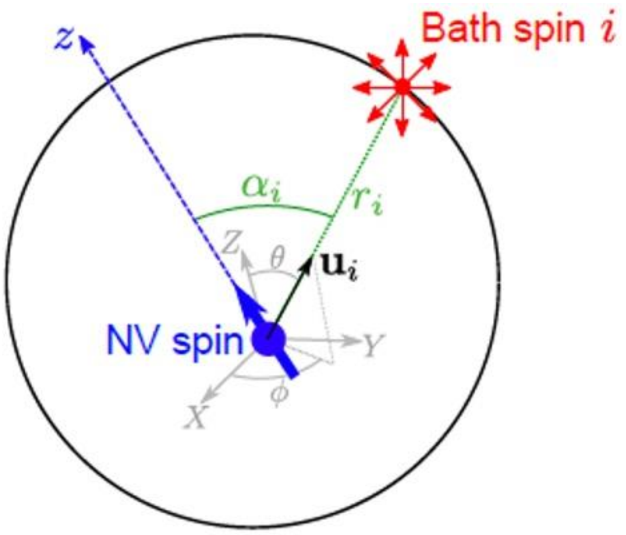

Fig. S14.

The schematic diagram of the model.

The determination of simulation parameters

a. The density and depth of NV centers: The same bulk diamond as used in other work has been adopted in our experiments (*56*). The density and depth of NV centers within the bulk diamond are 1000/um^2 and 5 nm, respectively.

b. The density and depth of Gd^{3+} molecules:

The initial z-location of the Gd^{3+} is determined by the Flory model of the PEG. For the PEG we use (Mw: ~1000 g/mol), the Flory radius of the PEG is $R_F = N^{\frac{3}{5}} \cdot l \approx 2.25nm$, if we also take the radius of Gd^{3+} molecule (about 0.51 nm) into consideration, the Gd^{3+} molecule is 2.76 nm away from the diamond surface at their free state. Based on the T_1 value we measured after complete force-responsive polymer modified diamond, the Gd^{3+} molecule density is set to 9000/μm^2 in order to match the experimental data.

The effective interaction range between Gd^{3+} molecules and NV centers

For a single NV center, we need to consider the influence of adjacent Gd^{3+} molecules. In the calculation, we take the Gd^{3+} molecules inside a circle region around NV centers into consideration. We call the region effective interaction range. Based on the calculation (Fig. S15), for a single NV center, if the interaction range is larger than the effective interaction range, the additional Gd^{3+} will not influence the longitudinal relaxation process of the NV centers. Based on this, for each NV centers, we only consider influence of the Gd^{3+} molecules in the effective interaction range of the NV centers.

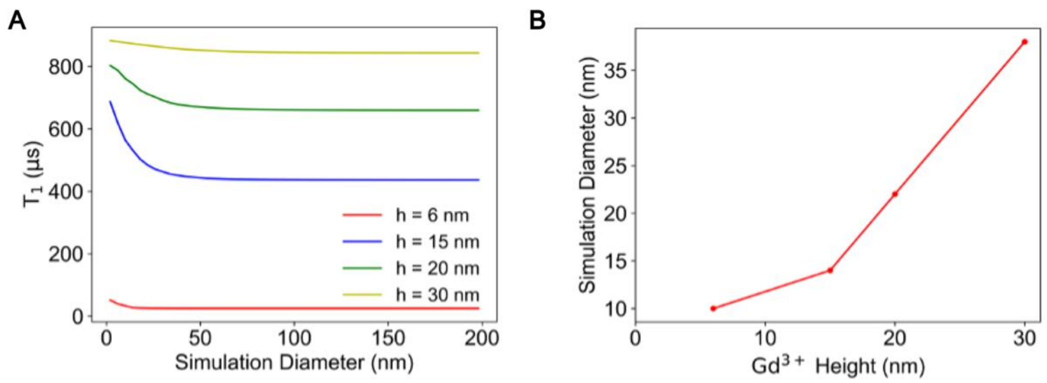

Fig. S15.

Determination of the proper simulation parameters. (A) T_1 change as the simulation diameter increases. **(B)** Effective interaction range changes with the height of Gd^{3+}.

The PEG model:

a. Theory model and experiment data for PEG

For the extension process of the PEG, the Worm-Like Chain model (*55*) is suitable to describe it. The previous study shows that the result of the experiment performed in PBS buffer, which is the same buffer we use in our experiment, can be well described by the model (*85*).

To check the suitability of the WLC model in our current work, the single-molecule force spectroscopy measurements for PEG molecules using AFM (JPK NanoWizard 4) have been conducted. The experiment was performed in the PBS buffer, which is setted as the same as the environment used in cellular force experiments. The overall experimental setup and procedure were demonstrated in Fig. S16A. After the experiments, the real tip-to-surface distance was obtained by subtracting the cantilever bending from the Z position of the cantilever. The zero point of the force was determined from the untouched part in the beginning of the force curve. The apparent length of the PEG molecules varied as a result of its length distribution. Therefore, the length of the force curve was normalized at the length of a 130 pN certain force (The same method was used in Ferry Kienberger et al., Single Mol. 2000 (*85*). Fig. S16B showed the data distribution and the simulation result of the WLC model. As the experimentally measured data could be well fitted with WLC model, we can conclude that the WLC model is suitable to the current work.

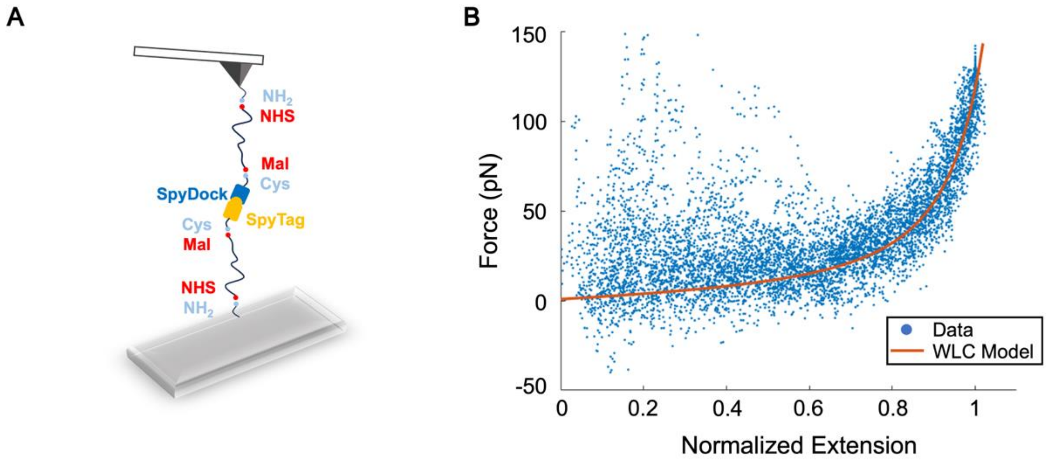

Fig. S16.

The calibration of the force-distance relationship of polyethylene glycol molecules based on the AFM single-molecule force spectroscopy. (A) Schematic of the single molecule force spectrum experiment of the PEG. **(B)** Force spectroscopy data and WLC model of the PEG molecules. The blue dot is the measured force spectroscopy data, and the line is the fitting of WLC model. The WLC model is expressed as $F = \frac{k_B T}{L_p}\left[\frac{1}{4(1-z/L_0)^2} - \frac{1}{4} + \frac{z}{L_0}\right]$

b. The PEG in our experiment

In our experiment, the molecular weight of the PEG we use is 1000, and the Persistence Length of PEG is about 3.7 Å (*85*. For PEG (Mw: ~1000 g/mol), the number of subunits is 22.3, the net length of PEG is 0.278 nm ~ 0.358 nm, then the Counter length of the PEG (Mw: ~1000 g/mol) is 6.20 nm ~ 7.98 nm. Here, we choose 7.68 nm. Based on this, the relationship between force and extension can be theoretically described (Fig. S17).

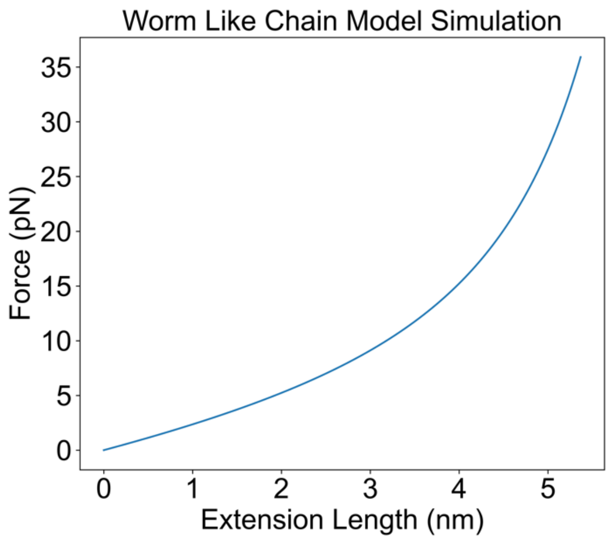

Fig. S17.

Force-Extension relationship of PEG (Mw: ~1000 g/mol) based on Worm-Like Chain model.

c. Flory model of the PEG

When PEG is in a good solution, it can be described by the Flory model (*86*). The free length of it is Flory radius, which is:

$$R_F = N^{\frac{3}{5}} \cdot l$$

For the PEG we use in this experiment, the Flory radius is 2.25 nm, which means that when there is no external force applied on PEG, the extension of it is 2.25 nm.

Evaluate the cytotoxicity of the microwave in living cells

To check the effects of microwave, we have used the standard live/dead ASSAY to evaluate the cytotoxicity of the applied microwave (parameters like power, duration being similar to the settings used in our typical T_1 measurement) in NIH 3T3 cells. We found that the viability of the cells treated with microwave shows no difference with the control experiment (without microwave treatment), as shown in Fig. S18. Therefore, the side effects on the cells caused by the microwave in current study are negligible.

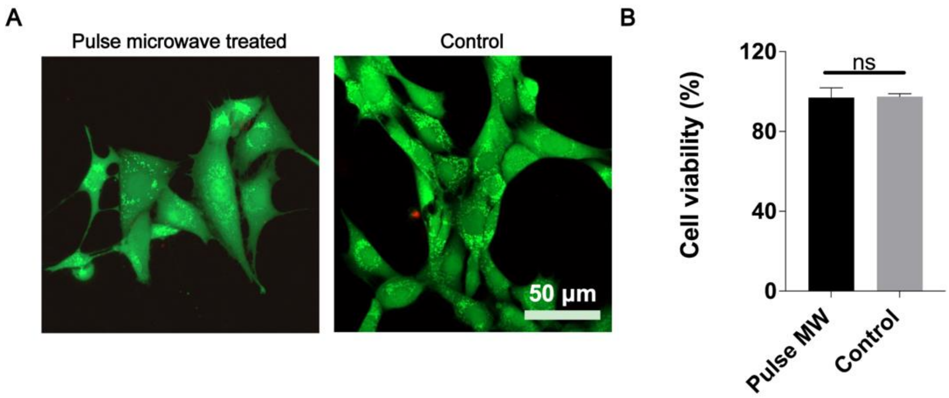

Fig. S18.

Investigating the influence of applied microwave on living cells. (A) Live/dead stain assay (Thermo, L3224) was used to view the NIH 3T3 cells treated with/without microwave. Green (calcein-AM, living cell), red (ethidium homodimer-1, dead cell). **(B)** The quantification of cell viability with/without microwave treatments (n=5, three technical replicates, p values were obtained by one-way ANOVA followed by Tukey's post hoc test, mean with standard deviation (S.D)).

REFERENCES AND NOTES

1. C. A. DeForest, D. A. Tirrell, A photoreversible protein-patterning approach for guiding stem cell fate in three-dimensional gels. *Nat. Mater.* **14**, 523–531 (2015).

2. P. Hou, Y. Li, X. Zhang, C. Liu, J. Guan, H. Li, T. Zhao, J. Ye, W. Yang, K. Liu, J. Ge, J. Xu, Q. Zhang, Y. Zhao, H. Deng, Pluripotent stem cells induced from mouse somatic cells by small-molecule compounds. *Science* **341**, 651–654 (2013).

3. Y. Ma, M. Lin, G. Huang, Y. Li, S. Wang, G. Bai, T. J. Lu, F. Xu, 3D spatiotemporal mechanical microenvironment: A hydrogel-based platform for guiding stem cell fate. *Adv. Mater.* **30**, e1705911 (2018).

4. F. Serwane, A. Mongera, P. Rowghanian, D. A. Kealhofer, A. A. Lucio, Z. M. Hockenbery, O. Campàs, In vivo quantification of spatially varying mechanical properties in developing tissues. *Nat. Mater.* **14**, 181–186 (2017).

5. A. Mongera, P. Rowghanian, H. J. Gustafson, E. Shelton, D. A. Kealhofer, E. K. Carn, F. Serwane, A. A. Lucio, J. Giammona, O. Campàs, A fluid-to-solid jamming transition underlies vertebrate body axis elongation. *Nature* **561**, 401–405 (2018).

6. D. E. Ingber, Mechanosensation through integrins: Cells act locally but think globally. *Proc. Natl. Acad. Sci. U.S.A.* **100**, 1472–1474 (2003).

7. D. E. Discher, P. Janmey, Y. L. Wang, Tissue cells feel and respond to the stiffness of their substrate. *Science* **310**, 1139–1143 (2005).

8. B. D. Hoffman, C. Grashoff, M. A. Schwartz, Dynamic molecular processes mediate cellular mechanotransduction. *Nature* **475**, 316–323 (2011).

9. H. Li, C. Zhang, Y. Hu, P. Liu, F. Sun, W. Chen, X. Zhang, J. Ma, W. Wang, L. Wang, P. Wu, Z. Liu, A reversible shearing DNA probe for visualizing mechanically strong receptors in living cells. *Nat. Cell Biol.* **23**, 642–651 (2021).

10. Y. Liu, K. Galior, V. P.-Y. Ma, K. Salaita, Molecular tension probes for imaging forces at the cell surface. *Acc. Chem. Res.* **50**, 2915–2924 (2017).

11. V. F. Fiore, M. Krajnc, F. G. Quiroz, J. Levorse, H. A. Pasolli, S. Y. Shvartsman, E. Fuchs, Mechanics of a multilayer epithelium instruct tumour architecture and function. *Nature* **585**, 433–439 (2020).

12. W. J. Polacheck, C. S. Chen, Measuring cell-generated forces: A guide to the available tools. *Nat. Mater.* **13**, 415–423 (2016).

13. A. Beaussart, S. El-Kirat-Chatel, R. M. A. Sullan, D. Alsteens, P. Herman, S. Derclaye, Y. F. Dufrêne, Quantifying the forces guiding microbial cell adhesion using single-cell force spectroscopy. *Nat. Protoc.* **9**, 1049–1055 (2014).

14. Y. Chang, Z. Liu, Y. Zhang, K. Galior, J. Yang, K. Salaita, A general approach for generating fluorescent probes to visualize piconewton forces at the cell surface. *J. Am. Chem. Soc.* **138**, 2901–2904 (2016).

15. X. Wang, T. Ha, Defining single molecular forces required to activate integrin and notch signaling. *Science* **340**, 991–994 (2013).

16. P. Roca-Cusachs, V. Conte, X. Trepat, Quantifying forces in cell biology. *Nat. Cell Biol.* **19**, 742–751 (2017).

17. S. J. Han, Y. Oak, A. Groisman, G. Danuser, Traction microscopy to identify force modulation in subresolution adhesions. *Nat. Mater.* **12**, 653–656 (2015).

18. R. Roy, S. Hohng, T. Ha, A practical guide to single-molecule FRET. *Nat. Methods* **5**, 507–516 (2008).

19. D. R. Stabley, C. Jurchenko, S. S. Marshall, K. S. Salaita, Visualizing mechanical tension across membrane receptors with a fluorescent sensor. *Nat. Mater.* **9**, 64–67 (2012).

20. I. Aharonovich, A. D. Greentree, S. Prawer, Diamond photonics. *Nat. Photon.* **5**, 397–405 (2011).

21. C. Dory, D. Vercruysse, K. Y. Yang, N. V. Sapra, A. E. Rugar, S. Sun, D. M. Lukin, A. Y. Piggott, J. L. Zhang, M. Radulaski, K. G. Lagoudakis, L. Su, J. Vučković, Inverse-designed diamond photonics. *Nat. Commun.* **10**, 3309 (2019).

22. N. Aslam, H. Zhou, E. K. Urbach, M. J. Turner, R. L. Walsworth, M. D. Lukin, H. Park, Quantum sensors for biomedical applications. *Nat. Rev. Phys.* **5**, 157–169 (2023).

23. L. Rondin, J. P. Tetienne, T. Hingant, J. F. Roch, P. Maletinsky, V. Jacques, Magnetometry with nitrogen-vacancy defects in diamond. *Rep. Prog. Phys.* **77**, 056503 (2014).

24. R. Li, F. Kong, P. Zhao, Z. Cheng, Z. Qin, M. Wang, Q. Zhang, P. Wang, Y. Wang, F. Shi, J. Du, Nanoscale electrometry based on a magnetic-field-resistant spin sensor. *Phys. Rev. Lett.* **124**, 247701 (2020).

25. S. Sotoma, C. P. Epperla, H.-C. Chang, Diamond nanothermometry. *ChemNanoMat* **4**, 15–27 (2018).

26. D. R. Glenn, D. B. Bucher, J. Lee, M. D. Lukin, H. Park, R. L. Walsworth, High-resolution magnetic resonance spectroscopy using a solid-state spin sensor. *Nature* **555**, 351–354 (2018).

27. K. S. Liu, A. Henning, M. W. Heindl, R. D. Allert, J. D. Bartl, I. D. Sharp, R. Rizzato, D. B. Bucher, Surface NMR using quantum sensors in diamond. *Proc. Natl. Acad. Sci. U.S.A.* **119**, e2111607119 (2022).

28. F. Shi, Q. Zhang, P. Wang, H. Sun, J. Wang, X. Rong, M. Chen, C. Ju, F. Reinhard, H. Chen, J. Wrachtrup, J. Wang, J. Du, Protein imaging. Single-protein spin resonance spectroscopy under ambient conditions. *Science* **347**, 1135–1138 (2015).

29. S. Sotoma, C. Zhong, J. C. Y. Kah, H. Yamashita, T. Plakhotnik, Y. Harada, M. Suzuki, In situ measurements of intracellular thermal conductivity using heater-thermometer hybrid diamond nanosensors. *Sci. Adv.* **7**, eabd7888 (2021).

30. G. Kucsko, P. C. Maurer, N. Y. Yao, M. Kubo, H. J. Noh, P. K. Lo, H. Park, M. D. Lukin, Nanometre-scale thermometry in a living cell. *Nature* **500**, 54–58 (2013).

31. M. Fujiwara, S. Sun, A. Dohms, Y. Nishimura, K. Suto, Y. Takezawa, K. Oshimi, L. Zhao, N. Sadzak, Y. Umehara, Y. Teki, N. Komatsu, O. Benson, Y. Shikano, E. Kage-Nakadai, Real-time nanodiamond thermometry probing in vivo thermogenic responses. *Sci. Adv.* **6**, (2020).

32. D. A. Simpson, E. Morrisroe, J. M. McCoey, A. H. Lombard, D. C. Mendis, F. Treussart, L. T. Hall, S. Petrou, L. C. L. Hollenberg, Non-neurotoxic nanodiamond probes for intraneuronal temperature mapping. *ACS Nano* **11**, 12077–12086 (2017).

33. J. Choi, H. Zhou, R. Landig, H.-Y. Wu, X. Yu, S. E. Von Stetina, G. Kucsko, S. E. Mango, D. J. Needleman, A. D. T. Samuel, P. C. Maurer, H. Park, M. D. Lukin, Probing and manipulating embryogenesis via nanoscale thermometry and temperature control. *Proc. Natl. Acad. Sci. U.S.A.* **117**, 14636–14641 (2020).

34. X. Feng, W. H. Leong, K. Xia, C. F. Liu, G. Q. Liu, T. Rendler, J. Wrachtrup, R. B. Liu, Q. Li, Association of nanodiamond rotation dynamics with cell activities by translation-rotation tracking. *Nano Lett.* **21**, 3393–3400 (2021).

35. L. P. McGuinness, Y. Yan, A. Stacey, D. A. Simpson, L. T. Hall, D. Maclaurin, S. Prawer, P. Mulvaney, J. Wrachtrup, F. Caruso, R. E. Scholten, L. C. L. Hollenberg, Quantum measurement and orientation tracking of fluorescent nanodiamonds inside living cells. *Nat. Nanotechnol.* **6**, 358–363 (2011).

36. L. Nie, A. C. Nusantara, V. G. Damle, R. Sharmin, E. P. P. Evans, S. R. Hemelaar, K. J. van der Laan, R. Li, F. P. Perona Martinez, T. Vedelaar, M. Chipaux, R. Schirhagl, Quantum monitoring of cellular metabolic activities in single mitochondria. *Sci. Adv.* **7**, eabf0573 (2021).

37. L. Nie, A. C. Nusantara, V. G. Damle, M. V. Baranov, M. Chipaux, C. Reyes-San-Martin, T. Hamoh, C. P. Epperla, M. Guricova, P. Cigler, G. van den Bogaart, R. Schirhagl, Quantum sensing of free radicals in primary human dendritic cells. *Nano Lett.* **22**, 1818–1825 (2022).

38. C. Reyes-San-Martin, T. Hamoh, Y. Zhang, L. Berendse, C. Klijn, R. Li, A. E. Llumbet, A. Sigaeva, J. Kawałko, A. Mzyk, R. Schirhagl, Nanoscale MRI for selective labeling and localized free radical measurements in the acrosomes of single sperm cells. *ACS Nano* **16**, 10701–10710 (2022).

39. K. Wu, T. A. Vedelaar, V. G. Damle, A. Morita, J. Mougnaud, C. R. San Martin, Y. Zhang, D. P. I. van der Pol, H. Ende-Metselaar, I. Rodenhuis-Zybert, R. Schirhagl, Applying NV center-based quantum sensing to study intracellular free radical response upon viral infections. *Redox Biol.* **52**, 102279 (2022).

40. K. Wu, L. Nie, A. C. Nusantara, W. Woudstra, T. Vedelaar, A. Sigaeva, R. Schirhagl, Diamond relaxometry as a tool to investigate the free radical dialogue between macrophages and bacteria. *ACS Nano* **17**, 1100–1111 (2023).

41. J. Barton, M. Gulka, J. Tarabek, Y. Mindarava, Z. Wang, J. Schimer, H. Raabova, J. Bednar, M. B. Plenio, F. Jelezko, M. Nesladek, P. Cigler, Nanoscale dynamic readout of a chemical redox process using radicals coupled with nitrogen-vacancy centers in nanodiamonds. *ACS Nano* **14**, 12938–12950 (2020).

42. V. Radu, J. C. Price, S. J. Levett, K. K. Narayanasamy, T. D. Bateman-Price, P. B. Wilson, M. L. Mather, Dynamic quantum sensing of paramagnetic species using nitrogen-vacancy centers in diamond. *ACS Sens.* **5**, 703–710 (2020).

43. T. Rendler, J. Neburkova, O. Zemek, J. Kotek, A. Zappe, Z. Chu, P. Cigler, J. Wrachtrup, Optical imaging of localized chemical events using programmable diamond quantum nanosensors. *Nat. Commun.* **8**, 14701 (2017).

44. L. T. Hall, G. C. G. Beart, E. A. Thomas, D. A. Simpson, L. P. McGuinness, J. H. Cole, J. H. Manton, R. E. Scholten, F. Jelezko, J. Wrachtrup, S. Petrou, L. C. L. Hollenberg, High spatial and temporal resolution wide-field imaging of neuron activity using quantum NV-diamond. *Sci. Rep.* **2**, 401 (2012).

45. J. F. Barry, M. J. Turner, J. M. Schloss, D. R. Glenn, Y. Song, M. D. Lukin, H. Park, R. L. Walsworth, Optical magnetic detection of single-neuron action potentials using quantum defects in diamond. *Proc. Natl. Acad. Sci. U.S.A.* **113**, 14133–14138 (2016).

46. C. Li, R. Soleyman, M. Kohandel, P. Cappellaro, SARS-CoV-2 quantum sensor based on nitrogen-vacancy centers in diamond. *Nano Lett.* **22**, 43–49 (2022).

47. R. W. de Gille, J. M. McCoey, L. T. Hall, J.-P. Tetienne, E. P. Malkemper, D. A. Keays, L. C. L. Hollenberg, D. A. Simpson, Quantum magnetic imaging of iron organelles within the pigeon cochlea. *Proc. Natl. Acad. Sci. U.S.A.* **118**, e2112749118 (2021).

48. M. Kayci, J. Fan, O. Bakirman, A. Herrmann, Multiplexed sensing of biomolecules with optically detected magnetic resonance of nitrogen-vacancy centers in diamond. *Proc. Natl. Acad. Sci. U.S.A.* **118**, e2112664118 (2021).

49. B. S. Miller, L. Bezinge, H. D. Gliddon, D. Huang, G. Dold, E. R. Gray, J. Heaney, P. J. Dobson, E. Nastouli, J. J. L. Morton, R. A. McKendry, Spin-enhanced nanodiamond biosensing for ultrasensitive diagnostics. *Nature* **587**, 588–593 (2020).

50. H. C. Davis, P. Ramesh, A. Bhatnagar, A. Lee-Gosselin, J. F. Barry, D. R. Glenn, R. L. Walsworth, M. G. Shapiro, Mapping the microscale origins of magnetic resonance image contrast with subcellular diamond magnetometry. *Nat. Commun.* **9**, 131 (2018).

51. S. Chen, W. Li, X. Zheng, P. Yu, P. Wang, Z. Sun, Y. Xu, D. Jiao, X. Ye, M. Cai, M. Shen, M. Wang, Q. Zhang, F. Kong, Y. Wang, J. He, H. Wei, F. Shi, J. Du, Immunomagnetic microscopy of tumor tissues using quantum sensors in diamond. *Proc. Natl. Acad. Sci. U.S.A.* **119**, e2118876119 (2022).

52. D. R. Glenn, K. Lee, H. Park, R. Weissleder, A. Yacoby, M. D. Lukin, H. Lee, R. L. Walsworth, C. B. Connolly, Single-cell magnetic imaging using a quantum diamond microscope. *Nat. Mater.* **12**, 736–738 (2015).

53. D. Le Sage, K. Arai, D. R. Glenn, S. J. DeVience, L. M. Pham, L. Rahn-Lee, M. D. Lukin, A. Yacoby, A. Komeili, R. L. Walsworth, Optical magnetic imaging of living cells. *Nature* **496**, 486–489 (2013).

54. P. Wang, S. Chen, M. Guo, S. Peng, M. Wang, M. Chen, W. Ma, R. Zhang, J. Su, X. Rong, F. Shi, T. Xu, J. Du, Nanoscale magnetic imaging of ferritins in a single cell. *Sci. Adv.* **5**, eaau8038 (2019).

55. C. Bouchiat, M. D. Wang, J. F. Allemand, T. Strick, S. M. Block, V. Croquette, Estimating the persistence length of a worm-like chain molecule from force-extension measurements. *Biophys. J.* **76**, 409–413 (1999).

56. S. Steinert, F. Ziem, L. T. Hall, A. Zappe, M. Schweikert, N. Götz, A. Aird, G. Balasubramanian, L. Hollenberg, J. Wrachtrup, Magnetic spin imaging under ambient conditions with sub-cellular resolution. *Nat. Commun.* **4**, 1607 (2013).

57. Q. Wei, T. Becherer, S. Angioletti-Uberti, J. Dzubiella, C. Wischke, A. T. Neffe, A. Lendlein, M. Ballauff, R. Haag, Protein interactions with polymer coatings and biomaterials. *Angew. Chem.* **53**, 8004–8031 (2014).

58. M. Xie, X. Yu, L. V. H. Rodgers, D. Xu, I. Chi-Durán, A. Toros, N. Quack, N. P. de Leon, P. C. Maurer, Biocompatible surface functionalization architecture for a diamond quantum sensor. *Proc. Natl. Acad. Sci. U.S.A.* **119**, e2114186119 (2022).

59. Q. Wei, R. Haag, Universal polymer coatings and their representative biomedical applications. *Mater. Horiz.* **2**, 567–577 (2015).

60. L. Yu, C. Cheng, Q. Ran, C. Schlaich, P.-L. M. Noeske, W. Li, Q. Wei, R. Haag, Bioinspired universal monolayer coatings by combining concepts from blood protein adsorption and mussel adhesion. *ACS Appl. Mater. Interfaces* **9**, 6624–6633 (2017).

61. J. Deng, C. Zhao, J. P. Spatz, Q. Wei, Nanopatterned adhesive, stretchable hydrogel to control ligand spacing and regulate cell spreading and migration. *ACS Nano* **11**, 8282–8291 (2017).

62. A. Ermakova, G. Pramanik, J. M. Cai, G. Algara-Siller, U. Kaiser, T. Weil, Y. K. Tzeng, H. C. Chang, L. P. McGuinness, M. B. Plenio, B. Naydenov, F. Jelezko, Detection of a few metallo-protein molecules using color centers in nanodiamonds. *Nano Lett.* **13**, 3305–3309 (2013).

63. M. Li, S. Jiang, J. Simon, D. Paßlick, M.-L. Frey, M. Wagner, V. Mailänder, D. Crespy, K. Landfester, Brush conformation of polyethylene glycol determines the stealth effect of nanocarriers in the low protein adsorption regime. *Nano Lett.* **21**, 1591–1598 (2021).

64. Q. Sun, Q. Wei, C. Zhao, How do the cells sense and respond to the microenvironment mechanics? *Chin. Sci. Bull.* **66**, 2303–2311 (2021).

65. Y. Nishimura, S. Shi, Q. Li, A. D. Bershadsky, V. Viasnoff, Crosstalk between myosin II and formin functions in the regulation of force generation and actomyosin dynamics in stress fibers. *Cells Dev.* **168**, 203736 (2021).

66. J. I. Lehtimäki, E. K. Rajakylä, S. Tojkander, P. Lappalainen, Generation of stress fibers through myosin-driven reorganization of the actin cortex. *eLife* **10**, e60710 (2021).

67. Q. Sun, Y. Hou, Z. Chu, Q. Wei, Soft overcomes the hard: Flexible materials adapt to cell adhesion to promote cell mechanotransduction. *Bioact. Mater.* **10**, 397–404 (2022).

68. C. Zhang, H. Zhu, X. Ren, B. Gao, B. Cheng, S. Liu, B. Sha, Z. Li, Z. Zhang, Y. Lv, H. Wang, H. Guo, T. J. Lu, F. Xu, G. M. Genin, M. Lin, Mechanics-driven nuclear localization of YAP can be reversed by N-cadherin ligation in mesenchymal stem cells. *Nat. Commun.* **12**, 6229 (2021).

69. J. Li, J. Di Russo, X. Hua, Z. Chu, J. P. Spatz, Q. Wei, Surface immobilized e-cadherin mimetic peptide regulates the adhesion and clustering of epithelial cells. *Adv. Healthc. Mater.* **8**, e1801384 (2019).

70. Y. Liu, K. Yehl, Y. Narui, K. Salaita, Tension sensing nanoparticles for mechano-imaging at the living/nonliving interface. *J. Am. Chem. Soc.* **135**, 5320–5323 (2013).

71. S. van de Linde, A. Löschberger, T. Klein, M. Heidbreder, S. Wolter, M. Heilemann, M. Sauer, Direct stochastic optical reconstruction microscopy with standard fluorescent probes. *Nat. Protoc.* **6**, 991–1009 (2011).

72. M. Lelek, M. T. Gyparaki, G. Beliu, F. Schueder, J. Griffié, S. Manley, R. Jungmann, M. Sauer, M. Lakadamyali, C. Zimmer, Single-molecule localization microscopy. *Nat. Rev. Methods Primers* **1**, 39 (2021).

73. R. Platzer, B. K. Rossboth, M. C. Schneider, E. Sevcsik, F. Baumgart, H. Stockinger, G. J. Schütz, J. B. Huppa, M. Brameshuber, Unscrambling fluorophore blinking for comprehensive cluster detection via photoactivated localization microscopy. *Nat. Commun.* **11**, 4993 (2020).

74. Z. Ge, H. Wei, F. Xu, Y. Gao, Z. Chu, H. K. H. So, E. Y. Lam, Millisecond autofocusing microscopy using neuromorphic event sensing. *Opt. Lasers Eng.* **160**, 107247 (2023).

75. A. Mzyk, A. Sigaeva, R. Schirhagl, Relaxometry with nitrogen vacancy (NV) centers in diamond. *Acc. Chem. Res.* **55**, 3572–3580 (2022).

76. K. J. Brown, E. Chartier, E. M. Sweet, D. A. Hopper, L. C. Bassett, Cleaning diamond surfaces using boiling acid treatment in a standard laboratory chemical hood. *J. Chem. Health Saf.* **26**, 40–44 (2019).

77. L. Yu, Y. Hou, C. Cheng, C. Schlaich, P.-L. M. Noeske, Q. Wei, R. Haag, High-antifouling polymer brush coatings on nonpolar surfaces via adsorption-cross-linking strategy. *ACS Appl. Mater. Interfaces* **9**, 44281–44292 (2017).

78. M. K. Moi, C. F. Meares, S. J. DeNardo, The peptide way to macrocyclic bifunctional chelating agents: Synthesis of 2-(p-nitrobenzyl)-1,4,7,10-tetraazacyclododecane-N,N',N'',N'''-tetraacetic acid and study of its yttrium (III) complex. *J. Am. Chem. Soc.* **110**, 6266–6267 (1988).

79. J. Liu, T. Xi, Enhanced anti-corrosion ability and biocompatibility of PLGA coatings on MgZnYNd alloy by BTSE-APTES pre-treatment for cardiovascular stent. *J. Mater. Sci. Technol.* **32**, 845–857 (2016).

80. H. Qi, Preparation of composite microporous silica membranes using TEOS and 1, 2-bis(triethoxysilyl)ethane as precursors for gas separation. *Chin. J. Chem. Eng.* **19**, 404–409 (2011).

81. Y. Wu, Y. Huang, H. Ma, A facile method for permanent and functional surface modification of poly(dimethylsiloxane). *J. Am. Chem. Soc.* **129**, 7226–7227 (2007).

82. J. P. Tetienne, T. Hingant, L. Rondin, A. Cavaillès, L. Mayer, G. Dantelle, T. Gacoin, J. Wrachtrup, J. F. Roch, V. Jacques, Spin relaxometry of single nitrogen-vacancy defects in diamond nanocrystals for magnetic noise sensing. *Phys. Rev. B* **87**, 235436 (2013).

83. C. Li, M. Chen, D. Lyzwa, P. Cappellaro, All-optical quantum sensing of rotational brownian motion of magnetic molecules. *Nano Lett.* **19**, 7342–7348 (2019).

84. X. Li, S. Zhang, H. Huang, L. Hu, F. Liu, Q. Wang, Unidirectional spin–orbit interaction induced by the line defect in monolayer transition metal dichalcogenides for high-performance devices. *Nano Lett.* **19**, 6005–6012 (2019).

85. F. Kienberger, V. P. Pastushenko, G. Kada, H. J. Gruber, C. Riener, H. Schindler, P. Hinterdorfer, Static and dynamical properties of single poly(ethylene glycol) molecules investigated by force spectroscopy. *Single Mol.* **1**, 123–128 (2000).

86. J. R. Fried, Conformation, solutions, and molecular weight, in *Polymer Science and Technology* (Pearson Education, 2003), chap. 3.
